# Neurological Insights into Sleep Disorders in Parkinson’s Disease

**DOI:** 10.3390/brainsci13081202

**Published:** 2023-08-14

**Authors:** Subramanian Thangaleela, Bhagavathi Sundaram Sivamaruthi, Periyanaina Kesika, Subramanian Mariappan, Subramanian Rashmi, Thiwanya Choeisoongnern, Phakkharawat Sittiprapaporn, Chaiyavat Chaiyasut

**Affiliations:** 1 Innovation Center for Holistic Health, Nutraceuticals, and Cosmeceuticals, Faculty of Pharmacy, Chiang Mai University, Chiang Mai 50200, Thailand; drthangaleela@gmail.com (S.T.); sivamaruthi.b@cmu.ac.th (B.S.S.); kesika.p@cmu.ac.th (P.K.);; 2 Office of Research Administration, Chiang Mai University, Chiang Mai 50200, Thailand; 3 PG and Research Department of Zoology, Yadava College, Madurai 625014, India; 4 Neuropsychological Research Laboratory, Neuroscience Research Center, School of Anti-Aging and Regenerative Medicine, Mae Fah Luang University, Bangkok 10110, Thailand

**Keywords:** Parkinson’s disease, sleep disorders, rapid eye movement, insomnia, excessive daytime sleepiness, sleep-related breathing disorders, circadian rhythm

## Abstract

Parkinson’s disease (PD) is a common multidimensional neurological disorder characterized by motor and non-motor features and is more prevalent in the elderly. Sleep disorders and cognitive disturbances are also significant characteristics of PD. Sleep is an important physiological process for normal human cognition and physical functioning. Sleep deprivation negatively impacts human physical, mental, and behavioral functions. Sleep disturbances include problems falling asleep, disturbances occurring during sleep, abnormal movements during sleep, insufficient sleep, and excessive sleep. The most recognizable and known sleep disorders, such as rapid-eye-movement behavior disorder (RBD), insomnia, excessive daytime sleepiness (EDS), restless legs syndrome (RLS), sleep-related breathing disorders (SRBDs), and circadian-rhythm-related sleep–wake disorders (CRSWDs), have been associated with PD. RBD and associated emotional disorders are common non-motor symptoms of PD. In individuals, sleep disorders and cognitive impairment are important prognostic factors for predicting progressing neurodegeneration and developing dementia conditions in PD. Studies have focused on RBD and its associated neurological changes and functional deficits in PD patients. Other risks, such as cognitive decline, anxiety, and depression, are related to RBD. Sleep-disorder diagnosis is challenging, especially in identifying the essential factors that disturb the sleep–wake cycle and the co-existence of other concomitant sleep issues, motor symptoms, and breathing disorders. Focusing on sleep patterns and their disturbances, including genetic and other neurochemical changes, helps us to better understand the central causes of sleep alterations and cognitive functions in PD patients. Relations between α-synuclein aggregation in the brain and gender differences in sleep disorders have been reported. The existing correlation between sleep disorders and levels of α-synuclein in the cerebrospinal fluid indicates the risk of progression of synucleinopathies. Multidirectional approaches are required to correlate sleep disorders and neuropsychiatric symptoms and diagnose sensitive biomarkers for neurodegeneration. The evaluation of sleep pattern disturbances and cognitive impairment may aid in the development of novel and effective treatments for PD.

## 1. Introduction

### 1.1. Parkinson’s Disease

Parkinson’s disease (PD) is a chronic progressive neurodegenerative condition characterized by the prominent death or loss of dopaminergic neurons in the substantia nigra pars compacta (SNPc) and the presence of intracellular protein α-synuclein (α-Syn) and ubiquitin-containing Lewy bodies. Defects in basal ganglia dopamine (DA) neurons produce motor difficulties [1] and non-motor complications like sleep disorders or wakefulness, neuropsychiatric and autonomic symptoms, pain, and other sensory difficulties [2]. PD affects many other brain regions, including the pigmented nuclei in the midbrain, brainstem, olfactory tubercle, cerebral cortex, and certain regions of the peripheral nervous system [3]. Regarding its neuropathology, PD causes a deficiency in DA neurons in the SNPc and the formation of Lewy bodies due to the aggregation of α-Syn protein in the cytoplasmic and axonal regions of neurons. 

In addition to DA neurons, PD pathology has been found to affect multiple other neuronal groups, including cholinergic, serotonergic, and adrenergic neurons in the brainstem and orexinergic neurons in the posterolateral hypothalamus, which play a crucial role in the development of sleep disorders in PD patients [4]. Early-onset PD is classified into juvenile PD and young-onset PD, which occurs between 21 and 40 years [5]. Although PD is common in both sexes, men are affected twice as much as women, showing the protective effects of female hormones [6]. The genetic bias of PD in the male population and exposure to environmental risks might cause high male preponderance in PD [5,6]. In addition to various other factors, gut microbiota plays an important role in PD. Gut microbial dysbiosis affects the gut lumen and produces inflammatory signals, which convey the signals to the central nervous system (CNS), disturbing the blood–brain barrier. Thus, the created neuroinflammatory signals can stimulate α-Syn accumulation and lead to development of PD [7]. 

The presence of PD in the family history could increase the probability of arousing a genetic form of PD [8]. PD is a non-monogenic disease caused by different genes, such as alpha-synuclein (*SNCA*), parkin 2 (*PARK2*), leucine-rich repeat kinase 2 (*LRRK2*), PTEN-induced putative kinase 1 (*PINK 1/PARK6*), ATPase type 13A2 (*ATP13A2*), parkin RBR E3 ubiquitin-protein ligase (*PRKN*), and glucocerebrosidase genes (*GBAs*) [9,10]. *SCNA* is the very first attributable gene in PD. Among PD patients, 5–10% were found to suffer from monogenic mutations in the genes *SCNA*, *LRRK2*, *VPS35*, *PINK1*, *DJ-1*, and *Parkin* [11]. Mutation in *SCNA* was the first reported genetic mutation and tends to cause early-onset PD [12]. The most common cause of inherited PD is due to a mutation in the *LRRK2* gene that causes late-onset PD, which was first identified in 2004 [11]. Mutation in the *GBA* gene is an important risk factor for PD. *GBA* carriers showed higher prevalence of dementia and cognitive deficits [13]. Other non-motor features, like anosmia, dysautonomia, depression, anxiety, hallucinations, and sleep disorders like RBD, are also prevalent in *GBA* carriers [14].

The hallmark clinical symptoms of PD include motor and non-motor impairment types that disturb normal daily living and quality of life. PD is a movement disorder associated with various motor symptoms, like bradykinesia, tremor, rigidity, slow movement, difficulty in walking and balance, and postural instability [15], and non-motor symptoms, such as hyposmia, constipation, urinary dysfunction, cognitive deficits, depression, pain, orthostatic hypotension, and sleep disturbances [16]. The presence of non-motor symptoms is the marker of the prodromal phase of PD. In addition, subtle motor signs involve a change in voice, decreased facial mobility, loss of finger dexterity, stooped posture, and reduced arm swing when walking [17]. 

The diagnosis of PD is mainly based on history and examination. Evaluating the history of the patient includes the evaluation of RBD, hyposmia, constipation, movement difficulties, and cognitive problems. The examination of PD includes examining the presence of motor symptoms [18]. Based on the diagnosis, PD patients are differentiated into four types, namely, idiopathic (or primary), symptomatic (or secondary), heredodegenerative, and multi-system degeneration parkinsonism patients [15]. The International Parkinson and Movement Disorder Society revised the Queens Square Brain Bank (QSBB) criteria for the clinical neurological examination of PD [19]. Various imaging techniques are now being used for diagnosing PD. In addition to structural magnetic resonance imaging (MRI), other novel methods, like neuromelanin imaging, quantitative susceptibility mapping, and visual assessment of dorsal STN hyperintensity, are also employed in diagnosing PD and differentiating it from other syndromes [20,21]. Sleep-related disturbances are among the frequent problems in PD. Sleep-related non-motor features of PD can be studied or diagnosed using Parkinson’s Disease Sleep Scale (PDSS). The severity of PD can be assessed using the Movement Disorder Society-Sponsored Unified Parkinson’s Disease Rating Scale (MDS-UPDRS) [22], Hoehn–Yahr Scale (H&Y Scale) [23], and Schwab–England scale (SES) [24].

Currently, no standard treatment is available for PD. Yet, standard procedures like oral-based pharmacotherapy or surgical alteration of brain regions affected by PD are in use. Different drugs are now available to treat motor impairment in PD and modify dopamine imbalances, including carbidopa/levodopa, pramipexole, and ropinirole [25,26]. Though levodopa is considered an effective drug against the early stages of PD, certain side effects, like levodopa-induced dyskinesias, are common [27]. Levodopa increases the dopamine level in the brain by triggering dopamine secretion. Other anticholinergic drugs help reduce tremors and muscle stiffness [28]. In the early stage of PD, dopamine agonists combined with levodopa are also used. Dopamine agonists can sometimes be used as monotherapy for PD to delay the need for levodopa [29]. Monoamine oxidase-B inhibitors are used to reduce PD symptoms. These inhibitors enhance the accumulation of dopamine in neurons. Generally, selegiline or deprenyl is the monoamine oxidase-B inhibitor used in combination with levodopa to reduce side effects [29]. Tolcapone is used in PD patients to reduce the use of levodopa. Amantadine blocks NMDA receptors and reduces levodopa-induced dyskinesias [30]. In more advanced methods, gene and stem cell therapy are now implemented for treating PD. Gene therapy in somatic cells can induce or inhibit target genes. Stem cell therapy is the administration of genetically modified stem cells into the brain part, where the cells multiply into healthy cells and produce dopamine [28]. Recently, ultrasound treatments [31] and rehabilitation therapies [32] have also been used as additional treatment methods to reduce complications in PD.

### 1.2. Sleep Disorders

Sleep is a human biological phenomenon alongside food, water, and air. In most humans, 20% to 40% of the day is spent sleeping [33]. Sleep problems in children and adolescents are also associated with behavioral, cognitive, and mood impairment. Insufficient sleep induces sleepiness, impaired learning, and problematic behavior. Maintaining sleep hygiene and specific treatments for sleep disorders may improve sleep and behavior [34]. When the biological need for sleep is influenced by various environmental and social pressures, sleep disturbances occur. Sleep problems are more common in older adults, typically presenting as difficulty in falling asleep and remaining asleep, and more daytime sleeping, as well as increased occurrence of insomnia, parasomnias, sleep apnea, and sleep–movement disorders [35]. Patients undergoing any medications for psychiatric disorders may also have severe sleep problems, which can lead to the development of neurodegenerative diseases [35]. Sleep disorders are associated with other mental disorders, like mood disorders, anxiety disorders, psychosis, and panic disorders, and neurological conditions, like parkinsonism, dementia, cerebral degenerative disorders, sleep-related epilepsy, and fatal familial insomnia [36,37,38,39,40]. Various other medical disorders are nocturnal cardiac ischemia, chronic obstructive pulmonary disease, sleep-related asthma, peptic ulcer, and irritable bowel syndrome, as well as fibromyalgia [41,42,43,44].

The International Classification of Sleep Disorders (ICSD) outlines seven major sleep disorders, including insomnia, sleep-related breathing disorders (SRBDs), circadian-rhythm sleep–wake disorders (CRSWDs), central hypersomnolence disorders, parasomnias, and sleep-related movement disorders and parasomnias [45]. From a behavioral perspective, sleep can be defined as the reversible state of perceptual disengagement or unresponsiveness towards the environment. The sleep regulation circuit involves genes; molecular signaling in neural circuits; and the process of transmission from the central nervous system to the peripheral organs, which controls movement, arousal, other autonomic functions, behavior, and cognition [46]. Wakefulness is also regulated by a complex neural circuitry mostly located in the reticular formation of the brainstem, termed ascending reticular activating system, which contains wake-responsible or wake-promoting neural cells groups like locus coeruleus adrenergic neurons in the basal forebrain; pedunculopontine cholinergic neurons in the tegmentum; serotonergic neurons in the raphe nucleus; dopaminergic neurons in the SN, VTA, and ventral periaqueductal gray matter; glutamatergic neurons in the mesencephalic reticular formation; orexinergic cells in the lateral hypothalamus; and histaminergic cells in the tuberomammillary nucleus [47,48,49]. These neuronal cell groups prepare a pack of activity-dependent metabolites to establish their functions throughout the brain and body. However, when it attains its threshold or critical level, this metabolic synthesis cycle starts a feedback loop function of disturbing the active behavioral state and initiating sleep by reducing the activity of wake-promoting neuronal cells [48]. Some sleep-inducing metabolites are adenosine, gamma-aminobutyric acid (GABA), prostaglandin, and cytokines IL-1β and TNF-α. Among them, GABAergic neurons in the hypothalamic preoptic area also inhibit the wake-promoting system’s signals [48]. Sleep and circadian rhythm are interconnected. Impairment in circadian rhythm disturbs sleep and may become a causative factor of neurodegenerative processes. The initial neurodegenerative processes affect the brain regions involved in sleep regulation and circadian rhythm, resulting in a disruption in sleep–wake cycle regulation [50].

Sleep disorders or disturbances are assessed using polysomnography (PSG), video-polysomnogram multiple sleep latency test (MSLT), actigraphy, maintenance of wakefulness test (MWT), nocturnal penile tumescence monitoring (NPT), electroencephalography (EEG), electrooculogram (EOG), submental electromyogram (EMG), electrocardiogram (ECG), and oximetry [51,52,53]. Treatment strategies include both pharmacological and non-pharmacological interventions. Methods of treatment vary according to the type of sleep disorder. Non-pharmacological methods mostly involve light therapies, practicing good sleep hygiene, and optimizing sleep. Pharmacological interventions include appropriate drugs, hypnotics, and antidepressants [53]. Sleep disorders are ubiquitous. Proper examination and on-time treatments are considered to be important in managing sleep disorders.

In this review, we emphasized the clinical characteristics of sleep disorders in PD; the genetic heterogeneity of PD; differences in sleep patterns in PD; other circadian-rhythm dysfunctions, REM, and RBD; associated neurological changes, symptoms, and diagnosis of sleep disorders; and treatment methods for PD. Sleep disturbances represent an important implication of PD, which may occur due to damage to the brain structures; other disruptions like depression and nocturnal immobility; and use of medications such as dopaminergic agents and antidepressants. Understanding the genetic background, circadian rhythm, and sleep patterns in PD conditions could lead to a better way of identifying and managing PD conditions earlier. This review provides an update on findings related to genetic background, etiology, symptoms, diagnosis, and treatment methods of PD-associated sleep disorders.

## 2. Sleep Disturbances in Parkinson’s Disease

**Figure 1:**
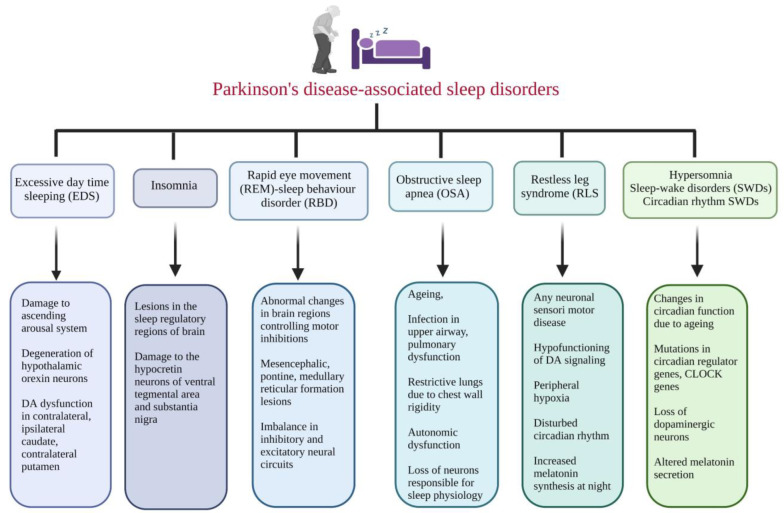
The illustration describes various sleep disorders associated with Parkinson’s disease and their causes and neurological changes. EDS: excessive daytime sleeping; DA: dopamine; REM: rapid eye movement; RBD: REM sleep behavior disorder; OSA: obstructive sleep apnea; RLS: restless legs syndrome; SWD: sleep–wake disorder; CLOCK: circadian locomotor output cycles kaput. (Figure created using BioRender.com; accessed on 27 June 2023).

Sleep is an important disease-modifying factor in PD. Sleep disturbance can cause altered sleep neural circuits, neurodegeneration, inflammatory reactions, impaired nocturnal brain oxygenation, and irregular proteostasis, which can provoke the development of α-synucleinopathies, further increasing the risk of PD [54]. The association between sleep disturbances and specific cognitive functions was evaluated in advanced PD patients. The results indicated that patients with sleep complaints performed worse than those without sleep complaints in terms of attention, reasoning, executive functions, and verbal fluency, but not memory. Also, PD-specific motor problems at night are correlated with neuropsychological dysfunctions in all studied cognitive domains, excluding memory. Additionally, no relationship was observed between daytime sleepiness and cognitive impairment [55]. Sleep disturbances in PD can also be due to other contributing factors, such as the side effects of dopaminergic drugs, other medications, comorbidities, genetic factors, lifestyle, and impulse control disorders [56]. In addition to disorders like bradykinesia, rigidity, tremors, and postural instability, and conditions like loss of dopaminergic neurons, sleep disturbances are seen in PD patients. The regulation and balance of sleep and wakefulness require the highly integrated functions of multiple brain regions and neurotransmitters. Parkinson’s disease-associated sleep disorders and their characteristics are illustrated in (Figure 1).

A wide range of sleep disorders, e.g., insomnia, sleep fragmentation, excessive daytime sleepiness (EDS), sleep-related breathing disorders (SRBDs), restless legs syndrome (RLS), nightmares, circadian-rhythm-related sleep–wake disorders (CRSWDs), obstructive sleep apnea (OSA), rapid eye movement (REM), and REM sleep behavior disorder (RBD), were observed in PD patients [57]. Visual hallucinations, psychosis, autonomic disturbances, dementia, and abnormal behaviors during sleep, such as dream enactments and excessive muscle twitching during REM sleep, are the characteristic features of RBD. PD patients with RBD also have visual hallucinations, psychosis, autonomic disturbances, and dementia [58]. Sleep disturbances like RBD are commonly considered a prodromal stage of neurodegeneration diseases like PD, Lewy body dementia (LBD), and multi-system atrophy [59]. The evaluation of early signs, such as sleep disturbances, especially RBD-like symptoms, and CSF α-Syn levels, provides an understanding of the central causes, biomarkers, and strategies to develop effective treatment for PD. 

The relation between sleep disturbance and α-Syn levels in cerebrospinal fluid (CSF) was measured in PD, prodromal PD, and healthy subjects. The study showed that sleep disturbance was high in prodromal PD, followed by PD and healthy subjects. The CSF α-Syn levels were significantly lower in PD subjects with RBD than in subjects with only PD [60]. Wang et al. investigated the associations of sleep disorders and CSF α-Syn levels among healthy controls, prodromal PD patients, and early PD patients. Their study demonstrated that sleep disorders lowered CSF α-Syn levels, with reduced sensorimotor function and impaired motor function. It has been hypothesized that PD-RBD subjects show increased neurophysiological abnormalities compared with PD patients without RBD [61]. Mutation in *GBA1* variants has effects on CSF α-Syn profiles. Hence, CSF α-Syn acts as a biomarker depending on mutation severity. The results of a large PD cohort study revealed that CSF α-Syn levels were reduced with respect to *GBA1* mutation. In addition to *GBA1* mutation, age is an important factor, where older age is associated with increased CSF α-Syn levels [62]. 

Depending upon the reported symptoms of sleep disorders, sleep behavior differs according to gender differences. Studying the importance of gender differences in sleep disorders can help improve the diagnosis, treatment, and prevention of sleep disorders and comorbid conditions [63]. Various factors, including hormonal and physical changes in a woman’s lifespan, can influence her sleep health. Certain sleep disorders like OSA and insomnia are more prevalent in women during specific periods [64]. On the other hand, narcolepsy, REM, and RBD are predominant in men, and the risk of RLS is double in women compared with men [63].

### 2.1. Excessive Daytime Sleepiness (EDS)

Sleep disturbances and wakefulness are the most common non-motor symptoms of PD. EDS affects 16 to 55% of PD patients, and the severity of EDS increases with disease duration and severity [65]. EDS is the second most prevalent, troublesome sleep-disorder symptom in PD, and it could be the preclinical marker for the development of PD [66]. EDS can be defined as the sleep trend or falling asleep excessively during various activities like reading, eating, and other circumstances. The progression of EDS equals the rate of progression of neurodegeneration [67]. EDS can be a prodromal risk factor for further neurodegeneration and increased risk of PD. EDS in PD showed a correlation with alterations in cerebral regions, such as the hypothalamus and brainstem regions; damage to the ascending arousal system; and changes in neurotransmitter and neuropeptide balances, especially GABAergic, orexinergic, and serotonergic systems [68,69]. 

In a study, EDS was measured in baseline PD patients using the Epworth Sleepiness Scale (ESS) for up to 3 years. The results indicated that the ESS score was increased from baseline to the third year in the PD group, with no changes in healthy controls. Conclusively, it was found that EDS significantly increased over time in PD relevant to the dosage of dopaminergic therapy but remained unchanged in healthy controls. A 123I ioflupane dopamine transporter imaging (DaTscan) study showed that the biological correlates of PD and EDS exhibited major dopaminergic dysfunction in brain regions like contralateral and ipsilateral caudate, and contralateral putamen compared with PD patients without EDS [70]. 

IPD patients were evaluated for nocturnal disturbance, EDS, and RBD symptoms with neuropsychological testing and self-report questionnaires. The study results demonstrated that patients with EDS showed significantly poor working memory, and RBD patients showed poor working memory and verbal fluency. Brain regions like medial temporal regions and subcortical regions were found to be associated with nocturnal disturbances, memory consolidation, and slow processing speed [71]. 

### 2.2. Insomnia

Insomnia was reported in 80% of PD patients, with difficulty in falling and staying asleep, and poor sleep quality. The frequency of insomnia is directly proportional to the advancement of motor stages in PD [45,72]. Primary insomnia and secondary insomnia develop due to depression, nocturnal worsening, and motor and non-motor dysfunctions [54]. PD insomnia and hyposomnia pathophysiology involves tremors, RLS, night-time cramps, dystonia, dyskinesia, and non-motor symptoms like psychiatric and autonomic dysfunctions [69]. The studied pathophysiological factors of insomnia include circadian-rhythm disruption, mutation in circadian locomotor output cycles kaput (*CLOCK*) genes, and neurochemical imbalances. In addition, disturbances in cortisol secretion and lesions in the sleep regulatory systems of the brain also cause insomnia [73]. Insomnia in PD might occur for various reasons, including the neurodegeneration of sleep regulation centers like the hypothalamus and brain stem, and continuous medications like dopaminergic drugs [74]. A longitudinal follow-up study suggested that the frequency of insomnia subtypes was changed in early PD patients. Also, the frequency of sleep-maintenance problems increased with dopamine agonists [75].

Insomnia in PD has been shown to increase cognitive decline and mental illness and to exert negative impacts on the health of individuals. Basal ganglia neural circuits and dopaminergic neurons in the SN and VTA are involved in sleep regulation. The effects of lesions in the basal ganglia and SN on sleep were evaluated in PD patients and animal models of PD [76]. Given the involvement of the basal ganglia in sleep maintenance, recent techniques use basal ganglia neuromodulation to ameliorate PD insomnia [76]. 

### 2.3. Rapid-Eye-Movement (REM) Sleep Behavior Disorder (RBD)

RBD is one of the prodromal symptoms of PD. RBD showed visual hallucinations, dream enactments, muscle twitching during REM sleep, psychosis, autonomic disturbances, and dementia [57]. Abnormal dream enactments characterize RBD during REM sleep with activities such as punching, waving, swinging wildly, or jumping out of bed [77]. Besides dream-enactment behaviors, clinical characteristics like severe cognitive and motor impairment, higher sleeplessness, and hallucinations were also observed in RBD [78]. Any changes in the brain stem regions that control motor inhibitions during REM sleep could result in RBD. Mesencephalic, pontine, or medullary reticular lesions were observed in animal models in REM sleep without atonia [77]. 

Studies in PD patients with RBD and cognitive deficits showed functional disturbances in the dorsolateral prefrontal cortex and posterior cortical regions [79,80]. RBD with non-motor symptoms and constipation are the predictors of the conversion of RBD into parkinsonism [81]. REM without atonia, with increased sustained and intermittent electromyographic (EMG) activity, is the hallmark neurophysiological symptom of RBD. Any impairment or imbalance in the neural circuits that control the excitatory and inhibitory signals results in episodic sleep disturbances in RBD. REM without muscle atonia can be differentiated into iRBD and secondary RBD, which occurs in PD patients [54]. PD patients with the RBD phenotype were predominantly older males. They possessed akinetic–rigid dominant motor disease, autonomic dysfunction, increased falls, EDS, and increased risk of developing future dementia and visual hallucinations [82]. An analysis using psychiatric/clinical questionnaires and neuropsychological assessment in PD patients with probable RBD and healthy controls revealed that RBD affects 33–46% of PD patients and poses the risk of neuropsychological deficits such as poorer cognitive, functional, and emotional outcomes [83].

### 2.4. Obstructive Sleep Apnea (OSA)

**Figure 2:**
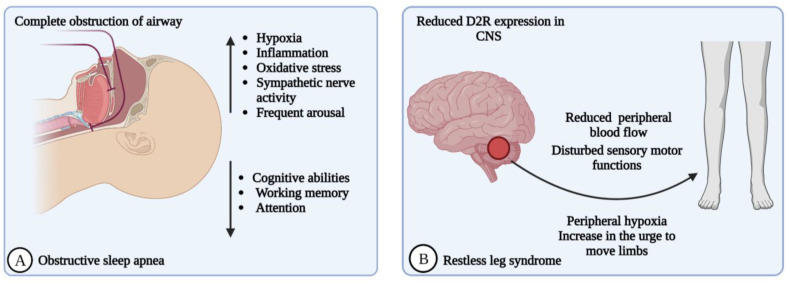
(**A**) Obstructive sleep apnea (OSA): The episodic breathing session and repeated obstructions in the upper airway worsen nocturnal respiration and sleep. OSA produces irregular respiratory patterns, hypoventilation, nocturnal worsened respiration, and oxidative stress due to the resaturation and desaturation of oxygen levels, and produces damage to dopaminergic neurons. (**B**) Restless legs syndrome (RLS): Hypo-functioning of dopamine signaling due to reduced dopamine subtype 2 receptor (D2R) expression in the CNS. Reduced peripheral blood flow causes peripheral hypoxia, which leads to urges to move legs and causes defects in neurological sensorimotor functions. (Figure created using BioRender.com; accessed on 27 June 2023).

OSA and PD coincide with one another. The incidence of OSA in PD or the PD-predisposing condition of OSA is high. Large-scale population follow-up studies describe the increased incidence of OSA in PD [84]. SRBDs are the least commonly studied sleep disturbances associated with PD. Patients with postencephalitic parkinsonism showed changes like irregular respiratory patterns, hypoventilation, and nocturnal respiration worsening. OSA is a common comorbidity, and obstructive, central, and mixed apnea types have been documented in PD [85]. OSA is characterized by episodic cessation of breathing due to partial (hypopnea) or complete (apnea) recurrent obstructions in the upper airway, resulting in periodic arrests in breathing during sleep (Figure 2A). These disturbances in breathing consequentially cause intermittent hypoxia and frequent arousal during OSA [86]. The symptoms of OSA are commonly associated with sleep apnea, such as cognitive impairment, sleepiness, nocturia, and snoring [56].

Repeated oxygen desaturation and resaturation that occur during sleep can result in the production of reactive oxygen species that initiate oxidative stress and certain molecular events that interfere with the cellular proteins, lipids, and mitochondrial functions that damage the dopaminergic neurons in the brain and produce neurodegeneration in PD [87]. Age is the major risk factor for the development of OSA. Other than age, infection in the upper airway, pulmonary dysfunction, and some PD-associated symptoms (including restrictive lungs due to chest-wall rigidity, postural instability, autonomic dysfunction, and loss of neurons in the brain sites responsible for sleep physiology) contribute to OSA [88].

Clinically, OSA could cause other sleep-related issues, like EDS, nocturia, non-refreshing sleep, and memory problems [86]. A recent meta-analysis stated the role of the severity of OSA in establishing cognitive disturbance in PD patients. PD patients with OSA scored significantly lower on the Montreal Cognitive Assessment (MoCA) and Mini-Mental State Examination (MMSE). The results suggest that OSA can worsen cognitive abilities like working memory, attention, and executive functions independently of PD-associated cognitive decline due to other factors, like sleep fragmentation, hypoxemia, neuroinflammation in the brain stem nuclei, and malfunction in certain brain regions [89]. Another meta-analysis revealed that OSA acts as a risk factor for PD. Chronic intermittent hypoxia due to OSA induces oxidative stress and inflammatory pathways, which result in PD pathophysiology [90]. 

Meng and colleagues studied the association between OSA and motor dysfunction, and the effect of OSA treatment. PD patients with OSA were treated with continuous positive airway pressure (CPAP), and motor symptoms were assessed using the Movement Disorder Society-Sponsored Unified Parkinson’s Disease Rating Scale (MDS-UPDRS) and Timed Up and Go (TUG) at 3, 6, and 12 months of follow-up. The results showed that PD-OSA individuals showed higher MDS-UPDRS scores at baseline and CPAP treatment stabilized the motor function over 12 months [84]. Upper-airway dysfunction was reported in some PD patients, which shows that laryngopharyngeal motor dysfunction is one of the factors that cause obstructive phenomena of upper-airway dysfunction in OSA-PD patients [91]. An observational study in 239 Chinese PD patients with and without OSA revealed certain characteristic features of the disease, such as age and male gender, which are the risk factors for OSA in PD. PD patients with RBD and higher levodopa equivalent doses showed a lower risk of developing OSA [92]. Certain studies in relation to sleep disorders in PD are given in Table 1. 

### 2.5. Restless Legs Syndrome (RLS)

RLS is a sleep–movement disorder, more frequent in PD patients, known as Willis–Ekbom syndrome, characterized by unpleasant sensations and uncontrollable urges to move legs, arms, and other body parts [105]. RLS is an overwhelming urge to move the body to resolve uncomfortable feelings like creeping, tingling, crawling, pulling, or pain inside the limbs [106]. RLS is a sensorimotor neurological disease that may cause disturbances in sleep, sleep maintenance, and quality of life. RLS can be caused by various factors, including genetic, environmental, and medical factors [105]. Although the pathogenesis of RLS is not yet clear, a few theories postulate that one of the reasons for it is the involvement of dopamine, particularly the hypo-functioning of dopamine signaling [107] Another reason for RLS pathogenesis could be the reduced peripheral blood flow, which causes altered dopamine availability in the periphery. This peripheral hypoxia causes the urge to move legs or arms to improve tissue oxygenation [107] The dopamine neurotransmitter shares a link between the immune system and CNS mediated by peripheral dopamine receptors, a CNS dopamine function biomarker. Due to changes in blood dopamine concentrations, dopamine subtype 2 receptor (D2R) expression is reduced in monocytes and lymphocytes due to altered CNS expression. Reduced D2R expression creates insensitivity in monocytes and lymphocytes towards dopamine, a characteristic feature of RLS (Figure 2B) [107].

RLS and PLMSs have been investigated for decades, and it was confirmed that PD patients found to also have RLS showed early-morning dystonia, akathisia, neuropathic pain, nocturnal hypokinesia, nocturnal leg cramps, and biphasic dyskinesia [56]. RLS is an important sleep disturbance involving the circadian rhythm [108]. In RLS, the involvement of the circadian rhythm can be understood as increased sensorimotor symptoms during the night due to increased melatonin secretion, which inhibits dopamine synthesis in the CNS [109]. Also, RLS symptoms peak at night, when the core body temperature decreases, and vice versa during the day. This suggests the involvement of the circadian rhythm in RLS [110].

## 3. Neurological Changes in Sleep Disturbances

EDS is another of the most frequent symptoms of neurological diseases like PD, multiple sclerosis, and myotonic dystrophies. Neurological patients with EDS possess psychiatric symptoms, cognitive deficits, and increased severity of neurological disease [111]. EDS can be explained as inappropriate sleepiness during waking hours and one of the most-filed sleep complications in PD patients, and it determines the patient’s quality of life. 

**Figure 3:**
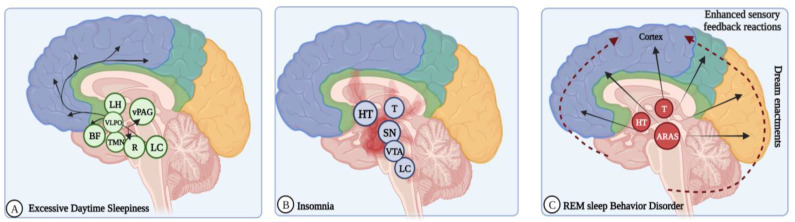
Schematic diagram showing key components of physiological and neurological changes in sleep disorders. (**A**) Excessive daytime sleeping (EDS): EDS occurs due to damage to the ascending arousal system (AAS), degeneration of hypothalamic orexin neurons, dopaminergic dysfunction, and increased dopaminergic therapy (DT) dosage. PD patients with EDS show changes in the AAS, including neurotransmitter systems like dopamine–ventral periaqueductal gray matter (vPAG), orexin–lateral hypothalamus, serotonin–raphe, noradrenaline–locus coeruleus (LC), histamine–tuberomammillary nucleus (TMN), acetylcholine, GABA–basal forebrain (BF). (**B**) Insomnia: Lesions in the regulatory sleep regions of the brain, like substantia nigra (SN), ventral tegmental area (VTA), and LC, cause disturbances in the sleep–wake cycle. (**C**). REM sleep behavior disorder (RBD): A common parasomnia due to loss of skeletal muscle atonia, changes in the brain stem regions controlling motor movements during REM sleep, or any impairment in the excitatory and inhibitory neural circuits; overactivation of the ascending reticular activating system (ARAS) causes abnormal motor behavior and dream enactments in REM sleep. (Figure created using BioRender.com; accessed on 27 June 2023).

EDS causes autonomic dysfunction, cognitive impairment, and psychosis. Neurodegeneration of the ascending arousal systems, dopaminergic medication, and nocturnal sleep disturbances are the etiological factors of EDS [112]. PD patients with EDS showed changes in the brain volume, white matter integrity, and cerebral metabolism [113] also in other regions, like the locus coeruleus, median raphe nucleus, ventral periaqueductal gray matter, tuberomammillary nucleus, basal forebrain, and lateral hypothalamus [72]. EDS also exhibits dopamine caudate denervation (Figure 3A) [68].

PD patients with EDS showed significant presynaptic dopaminergic dysfunction in the contralateral and ipsilateral caudate and the contralateral putamen [70]. Non-motor consequences were found in EDS, including autonomic dysfunction, depression, anxiety, and probable RBD, excluding cognitive dysfunction and motor severity. Neurodegeneration was observed in the brain stem areas responsible for alertness control, autonomic function, mood, and REM atonia. Recorded pathological signs include Lewy bodies, loss of adrenergic neurons in the locus coeruleus, serotonergic neurons in the raphe nuclei, and cholinergic neurons in the pedunculopontine nucleus [114]. 

Several physiological and psychological events may occur during sleep, including maintaining physiology, energy conservation, neurogenesis, brain development, and memory consolidation. Sleep is a complex process or behavior generated by the homeostatic combination of the brain and circadian process [67]. Hence, most neurological disorders have been associated with sleep disturbances. Sleep disorders are attributed as common comorbid conditions along with neurological diseases like PD, AD, epilepsy, and amyotrophic lateral sclerosis (ALS) [85].

Any precise mechanism behind the connections between sleep/wake cycles and the circadian rhythm has not yet been documented; however, complete generalizations have been made in research studies [115]. The sleep architecture is altered in PD due to disease-related changes, like degeneration of cholinergic neurons in the basal forebrain, brainstem, and pedunculopontine nucleus, and noradrenergic neurons in the locus coeruleus, resulting in the reduction in REM sleep and RBD. And the loss of serotonergic neurons in the raphe nucleus contributes to reduced slow-wave sleep [116]. RBD is caused by the degeneration of glutamatergic REM-ON and GABA/glycinergic REM-ON neurons in the sub-laterodorsal nucleus [117]. RBD is managed by the involvement of cholinergic and monoaminergic neurons in the pedunculopontine nuclei, laterodorsal tegmental nucleus, sub-laterodorsal nuclei, and locus coeruleus, as well as neural networks responsible for the limbic system and neocortex [118]. 

The monoaminergic fibers are w”red ’ack to the basal forebrain, ventral preoptic area, and cerebral cortex. Their firing states vary according to sleep/wake states [119]. The brain dynamically functions in different ways to promote complete transitions between sleep and wake states. Impairment in thalamocortical arousal and degenerative lesions in brainstem sleep/wakefulness and REM-sleep regulatory centers produce sleep disturbances like insomnia and EDS [120]. Wakefulness is endorsed by neurons in the midbrain, pons, and posterior hypothalamus that produce acetylcholine, norepinephrine, dopamine, serotonin, histamine, and orexin neurotransmitters (Figure 3B) [119].

A few decades ago, the discovery of the neuropeptides hypocretin-1 and hypocretin-2 changed our knowledge about sleep/wake regulation. The wake-active neuropeptides hypocretin-1 and hypocretin-2 are produced by the lateral hypothalamus, and hypocretin neurons also contain melanin-concentrating hormone, which is active during sleep and suppresses wakefulness by inhibiting the monoaminergic systems [121]. The serotonergic system plays a prominent role in maintaining the sleep–wake cycle. Any dysregulation in this cycle could result in sleep disturbances in PD. This was proven in animal models with reduced serotonin levels due to raphe nucleic lesions [122]. Coherently, another study stated that sleep deprivation models showed increased neuronal size and firing rates during wake time and reduced the expression of serotonin receptors [123]. 

A complex network of brain stem and cerebral cortex neurological pathways manages sleep and arousal. Sleep disturbances can mainly be caused by the degeneration of sleep regulation centers in the brain stem and thalamocortical regions [124]. The ascending reticular activating system (ARAS) maintains wakefulness via the dorsal and ventral pathways, mediated by acetylcholine, serotonin, noradrenaline, histamine, dopamine, and orexin neurotransmitters. Another branch of the ARAS includes the noradrenergic locus coeruleus, serotonergic dorsal and ventral nuclei, dopaminergic neurons in the ventrolateral periaqueductal gray matter, and histaminergic neurons in the tuberomammillary nucleus, which innervates the hypothalamus via a ventral route (Figure 3C) [115].

Cholinergic and GABAergic neurons in the basal forebrain send their projections throughout the cortex, hippocampus, and amygdala. These neurons display the highest firing rates during wakefulness and REM sleep and the lowest firing rates during non-REM sleep [125]. Monoaminergic neurons represented as REM-off cells were shown to often stop firing during REM sleep. Cholinergic, glutamatergic, and GABAergic neurons become highly active during REM sleep [126]. Mostly, the sleep-promoting neurons in the lateral hypothalamus are GABAergic. The inhibitory neurotransmitters GABA and galanin in the ventrolateral preoptic and median preoptic areas play an important function in sleep onset and help maintain sleep [127,128].

The occurrence of RBD in PD patients is linked with dopaminergic neurotransmission regions that are affected by dopamine imbalance [124]. Though the dopaminergic firing rate is static, extracellular dopamine concentration in regions like the striatum and prefrontal cortex is significantly elevated during wakefulness [129]. Dopamine release by the medial prefrontal cortex and nucleus accumbens was observed in sleep and wake states [130]. REM sleep maintains neuronal homeostasis in the brain; disturbance in REM sleep might impact brain excitability, new-synapse formation, neurogenesis, and memory consolidation, and even loss of REM can result in neurodegeneration. REM sleep also maintains neuronal integrity, noradrenaline levels, and a few housekeeping functions of the brain. Certain changes, such as enhanced functional activity in the putamen, thalamus, globus pallidus, cerebellum, pons, and sensorimotor cortex, as well as reduced functional activity in the lateral premotor cortex and parietal–occipital association regions, were observed in the PD brain [131]. The neuronal degeneration of the brain stem and thalamocortical pathways is an important factor in sleep disturbances. The degeneration of neurons in the lower brain stem nuclei, which has been connected with the dopaminergic VTA of the midbrain, was also noted as a pathological reason [124].

## 4. Circadian-Rhythm Dysfunction in PD

The circadian cycle or rhythm combines behavioral and psychological changes that function throughout life. The circadian rhythm plays a prominent part in maintaining the sleep–wake cycle, the secretion of hormones, glucose homeostasis, cardiovascular health, and regulation of body temperature and energy balance. The circadian cycle bidirectionally regulates the energy imbalance and metabolic processes associated with various metabolic diseases, like obesity, diabetes, and cardiovascular diseases [98].

The circadian cycle is bound to the light–dark cycle and the time duration of the local environment. The circadian cycle is managed by zeitgebers, which include external stimuli such as mealtimes, work timings, exercise, and light exposure [132]. The circadian cycle is a biological timekeeper, and it is regulated by the suprachiasmatic nucleus (SCN) present in the anterior hypothalamus, which comprises about 10,000 neurons. The SCN receives external stimuli signals like light, meals, and other schedules [133]. SCN neurons project into the sleep-regulating brain regions like the ventrolateral preoptic nucleus, lateral hypothalamus, and locus coeruleus.

**Figure 4:**
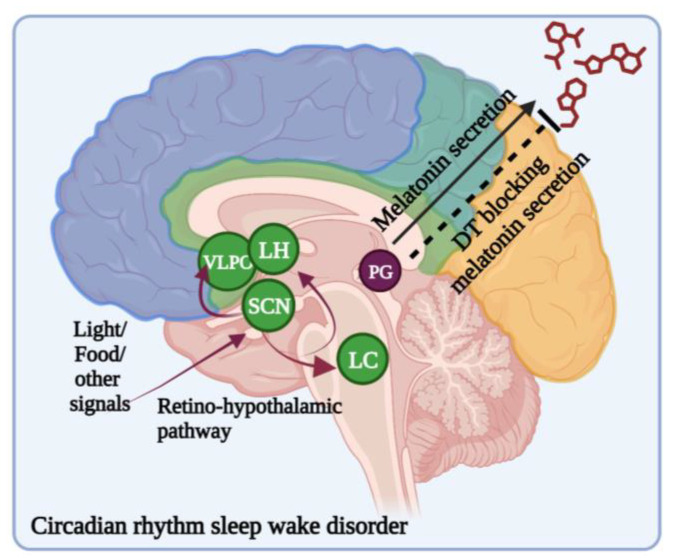
Circadian-rhythm sleep–wake disorders (CRSWDs): The SCN is the master clock that regulates circadian rhythm and signals. When light enters the retinal hypothalamic (RH) tract and reaches the SCN within the hypothalamus, the SCN signals the pineal gland to turn off melatonin production. Light-induced dopamine release or dopamine therapy-induced dopaminergic stimulation alters circadian-rhythm amplitudes, and mutations in circadian regulating genes cause changes in circadian-phase shift. (Figure created using BioRender.com; accessed on 27 June 2023).

Polymorphisms in the CLOCK gene cause sleep disorders [134]. A common form of circadian-rhythm sleep–wake disorder (CRSWD) is the delayed sleep–wake phase disorder (DSWPD), characterized by a delay in one’s sleep onset and wake time of 2 h or even more delayed compared with a normal individual [135]. DSWPD patients were found to have inherited mutations in period circadian regulator 3 (*PER3*) and cryptochrome *CRY1*, which can cause the lengthening of the circadian period [134]. The circadian cycle first starts with information relative to the incidence of light. Light information is detected by intrinsically photoreceptive retinal ganglion cells and passed onto the SCN (Figure 4) [136].

The encoded lIght informatio pas’es from the SCN to the hypothalamus, paraventricular nucleus (PVN), dorsomedial hypothalamic nucleus, sub-paraventricular zone, and medial preoptic nucleus (MPN). Most importantly, the hypothalamus is a key mediator in regulating the circadian rhythm [137]. Sleep–wake transitions are regulated by two processes, namely, the circadian (process C) and homeostatic processes (process S) [138]. Sleep rebound is said to be a corrective amount of sleep after a prolonged period of wakefulness. The homeostatic process is regulated by somnogens, which are adenosine molecules produced from the degradation of ATP accumulated during wake periods and reduced during sleep. Higher extracellular levels of somnogens promote the sleep state by inhibiting the ascending arousal system. On the contrary, the circadian pacemaker promotes wakefulness with the excitation of orexin neurons in the lateral hypothalamus [47]. 

Sleep onset, structure, and regulation are explained with the help of two process models. The combination of both homeostatic and circadian processes regulates sleep. The homeostatic sleep process, ‘process S’, increases during wake time and shuts down during sleep. The internal circadian clock, called ‘process C’, determines the alertness level [139]. The regular cycle of sleep and wakefulness is a highly complex function involving multiple brain areas and neurotransmitters. Dopamine plays a vital role in the circadian system’s function. In addition to sleep disturbances, the dysfunction of circadian function can also produce motor, autonomic, and cognitive warning signs in PD patients [140]. 

The sleep pattern is one of the most important factors disturbing the basal metabolic rate and energy regulation. There are two phases of sleep, REM and non-REM. The REM period is characterized by sympathetic nervous system activity and dreams. The metabolic rate is high during the REM phase [127]. Most neurodegenerative diseases have correlations with sleep disturbances and circadian-cycle dysfunction. Sleep–wake dysfunction is common with aging but becomes even worse in the case of neurodegenerative diseases [141]. Naturally, circadian rhythms change with aging; in the case of PD, circadian disruption and sleep–wake disturbances are more severe than in healthy older adults [4]. Circadian rhythm and sleep disturbances are closely related to PD and occur before motor symptoms. Disturbance in the circadian rhythm is linked to immune dysregulation, and disturbed protein balance in the brain leads to oxidative stress, which causes neurodegeneration [4]. An autopsy study stated that sleep–wake disturbances were associated with increased α-synuclein levels and PD pathology. This suggests that sleep disturbance is the risk factor for PD pathology in older adults [142]. It is still difficult to determine the correlation between circadian dysfunction and the occurrence of PD. However, certain studies suggest that circadian abnormalities precede the development of PD. There is a correlation among circadian abnormalities, sleep-related disturbances, and PD development. Thus, circadian rhythmicity could be a potential target or marker in PD management [4]. 

The behavioral and molecular level chInges in PD due to alterItions in or dysfunction of the circadian rhythm have been studied. Individuals with PD showed continuous disruption in their sleep–wake cycles and changes or reductions in the amplitude of circadian rhythmicity [4]. A large cohort study in community-dwelling older men without PD with 11 years of follow-up revealed that decreased circadian amplitude was associated with a higher risk of PD. Thus, reduced circadian rhythmicity helps in the early detection of PD and can be recognized as one of the prodromal features of PD [4]. 

The link between dopaminergic neuronal loss, inflammatory response in PD, and circadian dysregulation can explain the correlation between circadian regulation and PD. The relationship among circadian disruption, PD, and their consequences was evaluated in an experimental PD mouse model. Circadian-cycle-disrupted PD mice showed aggravated motor deficits with reduced capability of acquiring motor skills due to the loss of tyrosine hydroxylase and profound neuroinflammation. Neuroinflammatory reactions trigger the degeneration of the dopaminergic neuronal system, reproached for PD motor deficits [143].

Certain studies evidenced that PD patients receiving dopaminergic therapy (DT) presented with defects in SCN function and showed circadian dysregulation, like changes in the circadian phase and decreased melatonin levels at night [144,145]. Endo et al. studied the effect of chronic dopamine exposure on the function of the SCN and the expression of the circadian clock genes PER3, and Nr1d1 and -2 (nuclear receptor subfamily 1 group D member 1 and 2) at the single-neuron level in cultured mouse SCN and the clinical outcome of bright-light therapy against sleep problems in PD patients receiving DT. The results indicated that bright-light therapy improved sleep in DT-receiving PD patients. They also found that chronic dopaminergic stimulation attenuated SCN clock gene expression oscillations at the single-neuron level [93]. These results are consistent with the expression of the D1 dopamine receptor in the SCN, which modulates circadian-phase shift [146].

## 5. Genetic Heterogeneity of Sleep Disorders in Patients with PD

Sleep disorders are considered common non-motor symptoms mainly contributing to the pathophysiology of PD and prodromes of α-synucleinopathies. Several genetic variants contribute to sleep disturbance in PD and prodromal PD. The *SCNA, LRRK2, GBA*, and *PRKN* genes were reported to cause PD. The variants of these genes also contribute to the different clinical manifestations of PD [10]. Patients with *GBA* mutations were more likely to have the posture instability–gait difficulty phenotype than non-carriers [147]. *GBA* mutations also confer greater risk of developing dementia during the course of PD [94]. A huge single-cohort study in 1893 PD patients with *GBA* variants indicated that the risk of RBD development was significantly higher in PD patients with heterogenous *GBA* variants. *GBA* mutations significantly influence the age of onset, the severity of PD, and the motor phenotype in PD patients [16]. Patients with the *GBA* N370S variant had a higher RBD Screening Questionnaire score than patients with other variants [9]. The *GBA* E326k and T369M variants were not associated with RBD in PD patients. PD patients with *GBA* variants develop RBD more easily [62,148]. 

*GBA* mutations cause phenoconversion from the general RBD prodromal phenotype to clinical PD and dementia with Lewy bodies [149]. *GBA* mutations can occur in 2–23% of PD or LBD and in an early stage of PD onset. Postmortem studies showed that patients with RBD and those with *GBA* mutations had Lewy pathology [150,151]. *GBA p.E326k*, a highly pathogenic mutation, was also strongly associated with PD pathogenesis. RBD patients have genetic backgrounds similar to those of PD patients, like single-nucleotide polymorphisms in the *SCARB2* and *MAPT* regions. This signifies that RBD is associated with PD genetic markers, which may be helpful in the early detection of PD in patients with RBD [152]. 

A meta-analysis study showed that PD patients with heterozygous *GBA* variants are at high risk of developing RBD. PD patients with other *GBA* variants, like N370S and *L444P*, are at an even higher RBD risk than PD patients without these variants [153,154]. The *PRKN* gene is necessary for mitochondrial function in neuronal cells; its mutation is the most frequent in early-onset PD, and it was shown to cause the development of open mild RBD as examined using video-polysomnography [155]. The heterozygous *GBA1* mutant showed increased non-REM (NREM) sleep and reduced REM sleep durations. Mutation in *GBA1* increases the risk of idiopathic RBD (iRBD) and produces various structural changes in the neurocircuits of sleep. The conversion rate of α-synucleinopathy to neurodegeneration is higher in *GBA*-variant carriers [156]. 

The *LRRK2* gene, which codes for dardarin, also causes PD. A single mutation in Gly2019S causes 3 to 6% of familial PD and 2% of sporadic PD in Europe, and 37% of familial PD in Africa. PD patients with *LRRK2* mutations showed tremor-predominant parkinsonism, reduced cognitive deficits, and olfactory dysfunctions, but with more depression, anxiety, and irritability. Also, *LRRK2*-associated PD presents with sleep problems like reduced and fragmented sleep. But *LRRK2* patients are less likely to develop dementia than idiopathic PD (IPD) patients [100,157] *LRRK2*-mutation patients showed tremor-predominant parkinsonism, less cognition, and olfactory deficits, as well as more depression, anxiety, and irritability, compared with IPD patients. Also, sleep disturbances are more frequent in *LRRK2*-associated PD [158]. No significant differences in the risk of RBD were observed among patients with and without *LRRK2* mutations. *LRRK2* mutations with Gly2019S mutation caused milder non-motor symptoms, whereas the *LRRK2* G2385R mutation did not affect RBD risk [10]. *LRRK2*-PD patients frequently have complaints like RBD, poor sleep quality, sleep-onset insomnia, sleep fragmentation, and early awakening. EDS and RBD were observed after the onset of parkinsonism. RLS, periodic leg movements in sleeping (PLMSs), and OSA were not prominent in *LRRK2*-PD [159]. A full coding sequence analysis of *LRRK2* revealed that no pathogenic mutations were found to cause PD in RBD patients. Variant p.S1647T is associated with RBD risk and other haplotypes, *p.N551k-p.1398H-p.K1423k*, are associated with reduced RBD risk [160]. 

In another work, IPD patients (*n* = 11) and patients with two *parkin* mutations (*n* = 11) were assessed via sleep interviews, overnight video-polysomnography, and multiple sleep latency tests. The study results indicated that insomnia metrics and night-time and daytime sleepiness measures were similar among the patients, with non-significant differences. Parkin patients had sleep complaints like insomnia, RLS, and daytime sleepiness. The parkin sleep phenotype is similar to the IPD sleep phenotype except for RLS. OSA is predominant in Parkin patients as sleep apnea syndrome. Also, patients with abnormal sleepiness were found to have the Cys441 Arg mutation in the *PRKN* gene [161]. PD patients carrying homozygous and heterozygous *PRKN* variants showed severe RBD, RLS, or EDS [10]. Several studies have reported sleep disturbance due to disruption in the circadian rhythm. Sleep–wake-cycle disturbances in PD and Alzheimer’s disease (AD) are associated with genetic mutations. *Pink1* and *PRKN* gene mutations in drosophila showed sleep fragmentation [162].

Li et al. reported full sequencing and haplotype analyses of *MAPT* in PD, RBD, and dementia with Lewy bodies in RBD patients. The results revealed that *MAPT-H1* haplotypes were at increased risk of PD, and *MAPT-H2* variants had a protective effect against PD. The *MAPT H1/H2* haplotypes are not associated with RBD, suggesting the idea that RBD might have a different genetic reason from PD. Similarly, *LRRK2* pathogenic mutations are associated with PD, not RBD [163]. Mitochondrial genes also contribute to PD pathogenesis. Complex I of the respiratory chain was found to be defective in the SN. The nigral mitochondrial toxin 1-methyl-4-phenyl-1,2,3,6-tetrahydropyridine (MPTP) can cause acute parkinsonism, leading to the conclusion that mitochondrial genes are also responsible for PD pathogenesis [9]. 

Recently, Ling et al. studied the bidirectional causal relationship of PD-associated sleep phenotypes using the two-sample Mendelian randomization method. In this study, the authors selected 13 sleep-related phenotypes by searching published genome-wide association studies. They carried out a bidirectional MR study to understand the relationship between sleep-related phenotypes and the risk of age at onset of PD. Their results suggested a potential effect of PD on sleep and indicated that single-nucleotide polymorphisms are more frequent in insomnia patients with later-onset PD, but the analysis found no causal effect of poor sleep behavior on the onset of PD [164]. Other studies used Mendelian randomization analysis to evaluate either the sleep/wake cycle alterations’ causal relationship with neurodegenerative diseases like PD (considering age at onset), AD, and ALS [165], or the causal effects of genetically predicted OSA on neurodegenerative diseases like AD and PD [166]. These studies reported that there exists a significant causal relationship between OSA and PD or AD among the European population [163]. And sleep/wake cycle patterns act as possible risk factors and have a causal relationship with neurodegenerative diseases [165]. Highlights of genetic variants in sleep disorders and PD studies are listed in Table 2.

## 6. Evaluation of Sleep Disorders

### 6.1. Evaluation Scales and Screening Methods

Sleep disorders are the most observed and prevalent symptoms of PD. The demand for sleep studies is significantly enhanced due to heightened awareness of sleep disorders. Specific questionnaires were developed to evaluate PD patients with sleep disturbances. Parkinson’s Disease Sleep Scale (PDSS) [171], Scales for Outcomes in PD sleep (SCOPA-SLEEP), Pittsburgh Sleep Quality Index (PSQI), and ESS are used to validate patients with PD [172]. Polysomnography is a widely accepted gold-standard sleep study that has been in practice for decades to evaluate sleep disorders and their severity [173]. During this sleep study, the patient sleeps in a conducive environment having attached many electrodes all over their bodies with the help of a sleep technician. A sleep physician then analyzes the recorded signals. The sleep study can be carried out as either a full- or split-night study [174]. The graph obtained using polysomnography is known as a hypnogram and consists of three different parameters, EEG, EMG, and EOG, which describe the bioelectric activity of the brain, muscles, and eye movements, respectively [175]. Sleep depth, continuity, architecture, and distribution of sleep stages are characterized using polysomnographic analysis [176]. A polysomnography study is categorized into four levels depending on the symptomatology of the patient. Primarily, the study includes patients with RLS and cardiorespiratory diseases, periodic limb movement, behavioral disorders, parasomnias, sleep-related hypoventilation, neuromuscular conditions related to muscle weakness, usage of chronic medication of opioid drugs, history of stroke, and severe insomnia [177]. 

Sleep evaluation by sleep specialists includes primary information like initial history, sleep hygiene, and screening using the ESS. The ESS determines the patient’s daytime sleepiness. The ESS score is based on values from 0 to 24. When patients score equal to or more than 16 on the scale, they are considered to present with excessive sleepiness, and further investigations are suggested. The ESS score and polysomnography tests in patients help diagnose sleep disorders like EDS and OSA. To evaluate the background of sleep deprivation, other comorbid conditions, like RLS, emotional disorders, gastrointestinal disorders, and side effects of medications, must be assessed [163]. Home sleep-apnea testing is another newly generated sleep study to evaluate sleep apnea. This testing evaluates sleep with the help of peripheral arterial tone, oxygen desaturation, and heart rate [178]. As per international standards, different variables, such as light-off time, sleep latency, REM latency, total recording time, total sleep time, sleep efficiency, wake after sleep onset, wake after sleep offset, carbon dioxide level, and arousal from sleep, have been corroborated while interpreting sleep studies [174]. 

REM sleep shows faster electroencephalography activity, rapid horizontal eye movements, and hypotonia of skeletal muscles. With increased age, REM latency falls, and night-time sleep latency and wakefulness increase [175]. RBD diagnosis is based on the present diagnostic criteria, including IPD, RBD without atonia, and dream-enactment behaviors [115]. The exclusion criteria include vascular parkinsonism, drug-induced parkinsonism, and other metabolic causes of parkinsonism [179]. RBD diagnostic criteria include repeated episodes of highly variable sleep-related vocalization or complex motor behaviors documented with polysomnography and a history of dream enactments with movements or jerks, and they exclude other sleep-related movement disorders that mimic RBD [56]. Larger study populations without video-polysomnography readings can be screened with the help of questionnaires. A few other studies use actigraphy to evaluate subjects with iRBD and to differentiate iRBD from other motor activities during sleep. An actigraphy device is like a wristwatch, worn on the dominant hand, that monitors the activity and rest cycles of patients. Video analysis of actigraphy and clinical evaluation were found to be useful and reliable in screening iRBD in the general population [180]. Certain studies used RBD questionnaires such as the RBD Screening Questionnaire (RBDSQ) [181], Innsbruck RBD Inventory (RBD-I), Innsbruck RBD Summary Question [182], Hong Kong RBD Questionnaire (RBDQ-HK) [183], Mayo Sleep Questionnaire [184], and RBD Single-Question Screen (RBD-1Q) to validate RBD symptoms [185]. Recently, Mangone et al. used minor salivary gland biopsy (MSGB) to investigate the presence of PD using α-Syn immune staining in patients with IPD and iRBD. The study confirmed the findings, with the severity of lesions in SNPc being evaluated using neuromelanin-sensitive MRI [186]. 

The diagnosis of RLS is challenging and can be studied with the help of the RLS Rating Scale (RLSRS) and Non-Motor Symptom Questionnaire (NMSQ). RLS is diagnosed by evaluating symptom patterns like uncomfortable sensations, uncontrollable urge to move the legs, symptom worsening at rest, relief during movement, and exacerbations at night [187]. Sleep diagnoses have also been evaluated using screening scales like the Cleveland Sleep Habits Questionnaire (CSHQ) and STOP Questionnaires [188].

### 6.2. Neuroimaging Studies

Studying clinical features and pathological signs using neuroimaging may help in the early diagnosis of PD-associated sleep disorders and the timely initiation of treatments. Neuroimaging studies like diffusion-based magnetic resonance imaging (MRI) and studies like diffusion tensor imaging (DTI) are employed in PD patients with or without sleep disturbances to examine the alterations in cerebral structures. Functional MRI (fMRI) and DTI are advanced neuroimaging techniques used to study structural and functional changes in the preclinical stage of PD, which are usually difficult to determine with traditional MRI [189]. Brain imaging studies performed in PD-RBD patients and PD patients with cognitive impairment showed certain alterations in several brainstem nuclei regions and irregularities in serotonergic, dopaminergic, cholinergic, and noradrenergic neurotransmitter systems [190].

Transcranial magnetic stimulation is a neuroimaging tool used to study motor cortical excitability, neurotransmitter integrity, inhibition, and the facilitation of motor neuron circuitry in the brain [191]. With the help of transcranial magnetic stimulation, Bhattacharya and co-workers evaluated neurophysiological abnormalities in PD patients with and without RBD. The PD-RBD patients showed significantly strong inhibition of intracortical facilitation, glutamatergic transmission, and enhanced GABAergic transmission [57]. 

DTI is the most widely used imaging method for studying the integrity of white matter. The white matter can be evaluated by quantifying the diffusion orientation of water molecules in the brain using fractional anisotropy (FA). The FA method is clinically sensitive and captures microstructural alterations in the brain [192]. Any damage to neuronal cells or disruption in microstructural barriers produces assessable changes in the diffusion of water molecules and reduces the same in neurodegenerative conditions. FA values are generally reduced in neurodegenerative disorders. The reduced FA values are directly proportional to the severity of the disease. Thus, FA is a main contributive index for analyzing deep-brain structural abnormalities in PD [193]. DTI-FA applied with manual region-of-interest analysis helps identify the interconnections among microstructural changes in brain regions like the SN, thalamus, and hypothalamus in PD patients with sleep disorders [188]. 

Neuroimaging studies in RBD patients without any signs of parkinsonism or multi-system atrophy showed reduced striatal radiotracers’ binding values [124]. As per the European Association of Nuclear Medicine procedures, dopamine transporter imaging using dopamine transporter single-photon emission computed tomography (DAT-SPECT) can be used in PD and RBD patients [194]. Urso and their team investigated the neuroimaging marker to understand morphometric changes in the brainstem and other regions associated with RBD using the Magnetic Resonance Parkinsonism Index (MRPI) in de novo PD and iRBD patients. They found that PD patients with RBD had higher MRPI scores than patients without RBD. The MRPI scores also correlated with RBDSQ scores, which signifies that the MRPI can be used as a neuroimaging marker for RBD in PD patients [195]. PD patients with pRBD showed progressive loss of neurons in the Ponto mesencephalic tegmentum, medullary reticular formation, thalamus, hypothalamus, putamen, amygdala, and anterior cingulate cortex, as well as microstructural alterations in the cerebellar peduncles. Deformation-based morphometry analyses on T1-weighted MRI images are used to study differences in volumes of both the gray matter and white matter of the brain between PD patients with and without RBD [77]. 

### 6.3. Challenges in Diagnosis and Evaluation

The accurate diagnosis and screening of sleep disorders and PD-related sleep disorders remain challenging. Recognizing a sleep disorder in its initial stage is the most important step in treating the disorder. OSA is usually diagnosed by measuring the frequency of SDB events using overnight polysomnography. The Apnea–Hypopnea Index (AHI) denotes the number of sleep-apnea and -hypopnea events per hour. Severe OSA with an AHI index of over 30 is a risk factor for other clinical conditions, like diabetes, cancer, and cerebrovascular diseases, in adults [196]. OSA is mostly left undiagnosed due to symptoms such as snoring, and EDS is not recognized as an OSA sign by patients or primary care physicians. Appropriate diagnosis and treatment of OSA reduce the clinical and non-clinical consequences of the disease [196]. The main challenges in diagnosing sleep disorders in PD are identifying each sleep disorders, like EDS and RLS, and differentiating it from other cofounding factors, like medication effects, akathisia, nocturnal leg cramps, nocturnal hypokinesia, etc. [56]. Comprehensive diagnostic confirmation is required to clearly understand RBD. The greatest pitfall and challenge in diagnosing PD and sleep disorder consist in distinguishing PD from atypical Parkinsonian disorders like multi-system atrophy, progressive supranuclear palsy, and corticobasal degeneration [197,198]. This scenario will likely improve over the next decades. Advanced imaging markers and new approaches may enhance the differentiation of PD from other Parkinsonian disorders. Recent advancements in screening criteria, diagnostic markers, and genetic and imaging tests are making significant progress in the field of diagnosis and evaluation. Separate and precise differentiating diagnostic measures are required, as most sleep disorders and neurodegenerative diseases overlap with one another.

## 7. Treatment for PD-Associated Sleep Disorders

### 7.1. Non-Pharmacological Interventions

Various non-pharmacological approaches are available to treat and manage PD patients’ sleep disorders and circadian dysfunction. Along with a healthy diet, lifestyle practices that favor good sleep quality, along with exercises, avoiding daytime napping, constant bedtime, reduced caffeine intake (like chocolate), pain relievers with caffeine and other herbal supplements, and nicotine restriction of 6 h before sleep time, are important for patients, as they could improve sleep quality [199]. Physical activity, like high-intensity exercise, helps improve sleep efficiency and total sleep time [96]. Studies stated that traditional Chinese exercises like Tai Chi, Baduanjin, and Qigong improved sleep in PD patients [95,200,201]. Following the prescribed work–rest schedule with appropriate physical activity and receiving outdoor light may improve the sleep quality of PD patients [202]. Likely, cognitive behavioral therapy (CBT) could help to improve insomnia. Studies showed that CBT combined with BLT could significantly improve several sleep measures of insomnia severity in insomnia patients [203].

Bright-light therapy (BLT) was used to treat sleep disorders in Japanese PD patients who received DT. The study concluded that BLT improved their sleep states by improving circadian-phase shifts. Also, BLT could be the most promising treatment for improving sleep in PD patients by restoring circadian function [93]. A combination of BLT and CBT has beneficial effects and was found to produce long-term effects in young DSWPD patients. BLT and CBT helped correct the circadian rhythm and improved sleep time [204]. In sleep–wake disorders like DSWPD, advanced SWPD, and irregular SWPD, bright-light exposure stimulates the light–dark cycle, resulting in reduced daytime sleep and improved night-time sleep, circadian rhythm, and wakefulness in patients [134]. Numerous clinical studies indicate that BLT is effective in treating sleep disorders. For example, a randomized clinical trial was conducted on PD patients receiving DT. Dim-red-light therapy served as a control. The results stated that BLT patients experienced significantly improved EDS sleep fragmentation, sleep quality, and ease of falling asleep, and enhanced daily physical activity [205]. Dopaminergic therapy causes functional defects in the SCN circadian clock and abnormal melatonin secretion in DT-receiving patients [93,140]. To avoid complications, sleep disorders in PD patients are initially subjected to non-pharmacological interventions. 

Photobiomodulation (PBM) is another light therapeutic method using red to near-infrared light to heal, regenerate, and protect injured or degenerating brain tissue. The PBM mechanism depends on the enzyme cytochrome c oxidase of the mitochondrial respiratory chain. The mechanism behind PBM therapy is that cytochrome c oxidase activity is inhibited by the nitric oxide produced by damaged or hypoxic cells. The red or near-infrared photons of light dissociate nitric oxide and increase mitochondrial membrane’s potential, increasing oxygen consumption. More glucose is metabolized with increased oxygen, leading the mitochondria to produce more energy [206]. Transcranial PBM in the brain produced increased cerebral blood flow, and greater oxygen availability and consumption, and improved ATP synthesis and mitochondrial activity [206,207]. Intracranial application of red to infrared light improved locomotor activities and protected midbrain dopaminergic neurons in rodent and monkey models of PD [208]. Chromophores and heat-gated ion channels in neuronal cells absorb the photons and initiate cellular signaling pathways that produce positive effects on the brain [206]. 

Non-invasive brain stimulation approaches like repetitive transcranial magnetic stimulation and transcranial direct-current stimulation (tDCS) were found to have improved sleep quality in healthy elderly people [209]. Repetitive transcranial magnetic stimulation therapy also improved sleep scales, sleep efficiency, reduced sleep fragmentation, and nocturnal awakenings in PD and AD patients [210]. RBD patients experiencing sudden sleep attacks due to vivid dream content, orthostatic hypotension, and tremors cause injury to themselves or their bed partners. In such cases, non-pharmacological treatments can be used, including a bed with padded rails and a customized alarm [211]. Since many factors are involved in PD progression and motor/non-motor disabilities, complete treatments to modify or suppress the disease are currently unavailable [89]. The treatment of OSA impacts PD by attenuating disease progression and severity. Early interventions like continuous positive airway pressure treatment, lifestyle changes, and losing weight can be beneficial in reducing the incidence of PD in OSA patients [90]. Common treatments for CRSWDs are time therapy, light therapy, melatonin, hypnotic-drug therapy, and sleep-health education. 

### 7.2. Pharmacological Interventions

PD-associated sleep–wake disturbances significantly impact patients’ and their caregivers’ quality of daily life. Drugs such as safinamide and opicapone enhance the action of L-Dopa, and deep-brain stimulation methods have been developed to manage non-motor symptoms in PD patients [212,213]. Low doses of dopaminergic drugs help promote REM sleep and induce sleepiness; high doses reduce REM sleep and induce wakefulness [214]. The effect of levodopa/carbidopa intestinal gel (LCIG) infusion has been studied in PD patients. Sleep parameters were evaluated using PD-Sleep Scale Version-2 (PDSS-2) and ESS. LCIG infusion substantially developed nocturnal sleep and improved sleep quality, motor complications, and daily living [97]. The supplementation of levodopa/carbidopa/entacapone (LCE) improved sleep by improving motor and non-motor features in PD patients with sleep disturbance and motor fluctuations. Sleep onset, sleep maintenance, and RBD were especially significantly improved [215]. RBD patients were treated with 3 mg of melatonin for 4 weeks, resulting in slight to complete behavioral resolution [216]. In another study, longitudinal treatment with 6 mg of melatonin and 0.5 mg of clonazepam in RBD patients was evaluated. The result showed that both melatonin and clonazepam treatments reduced RBD behaviors. Melatonin-treated patients showed fewer adverse effects than clonazepam-treated individuals. Clonazepam-treated patients reported frequent side effects like unsteadiness and dizziness compared with the melatonin-treated group [217]. The short-term efficacy and safety of clonazepam in treating probable RBD in PD were studied by another team in a four-week randomized, double-blind, placebo-controlled trial with clonazepam (0.5 mg/day) at bedtime for RBD symptoms in PD patients. The clinical global impression–improvement score indicated that RBD symptoms improved in PD-RBD patients treated with both clonazepam and placebo [218]. This shows that to conclude on the efficacy of clonazepam, a firm study design may require large-scale randomized trials.

Hypnotic drugs, antipsychotics, and sedative antidepressants were used to treat various sleep disorders associated with neurodegenerative diseases. Supplementation with doxepin (10 mg/day) for six weeks significantly improved sleep quality and insomnia severity in PD patients [202]. Eszopiclone is a GABA-A-receptor agonist drug used in insomnia. PD patients with sleep-onset insomnia were treated with eszopiclone (3 mg for patients aged > 65 years, 2 mg for patients aged < 65 years and above) or placebo for 6 weeks. The patients treated with eszopiclone showed improved sleep quality and fewer awakenings without increasing total sleep time. But some side effects, like dizziness and daytime sleepiness, were observed in two patients [219]. The melatonin treatment of 3–10 mg/day for four weeks significantly reduced depressive scores and improved sleep continuity in DSWPD and depressive patients [220,221], and it was reported that melatonin treatment combined with a behavioral wake-up schedule helped reduce sleep-related disorders and improve sleep quality [221].

Modafinil is a wake-promoting agent used to treat many sleep disorders. The wake-promoting mechanism of modafinil involves the effects of monoaminergic systems like serotonin, dopamine, histamine, norepinephrine, and orexin in the brain. In addition to its effects on the sleep/wake system, modafinil also shows some neuroprotective effects. Modafinil enhances cellular metabolism and reduces free radicals in neurons, which increases ATP production. Adenosine, the breakdown product of ATP, promotes homeostatic sleep regulation [222]. 

Sleep-timing- and sleep-state-induced changes in muscle tone are regulated via adrenergic signaling. The dopamine autoreceptor agonist was found to blunt the wake-promoting efficacy of modafinil. This shows the involvement of dopamine-dependent adrenergic signaling in the mechanism of modafinil [223]. Modafinil and amphetamine treatments increased extracellular dopamine in narcoleptic dogs. In dopamine transporter-knock-out mice, NREM sleep was reduced, and wake time was increased. Dopamine transporter-knock-out mice remained unresponsive to the wake-promoting action of modafinil and amphetamine. This shows that the dopamine transporter is important for sleep regulation and the wake-promoting action of amphetamines and modafinil [224]. It was found that modafinil and amphetamine equipotentially induce dopamine release and increase wakefulness [225].

Caffeine is an adenosine-receptor antagonist that acts on A1 and A2A receptors and is functionally involved in brain-associated actions like sleep, arousal, and cognition. Caffeine is quickly absorbed into the small intestine, and with a highly variable half-life, caffeine improves daytime functioning and impacts sleep quality [226]. Caffeine has been used to lower EDS in PD patients. Caffeine is generally used to increase daytime alertness and improve motor benefits. A 6-week randomized controlled trial with caffeine (100–200 mg twice a day) showed improvements in EDS and other motor features, with a non-significant reduction in the ESS score and without any changes in sleep quality [227]. 

**Figure 5:**
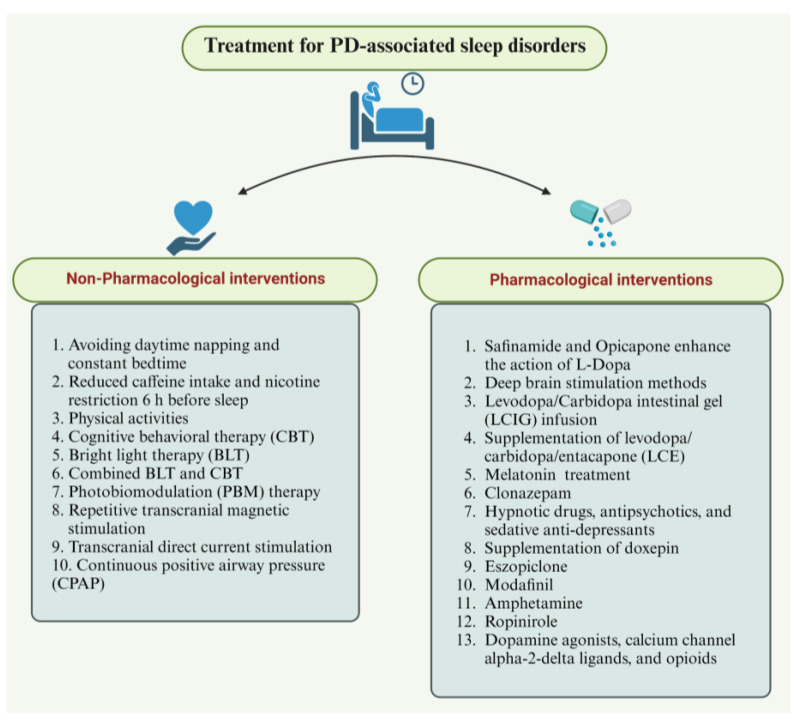
Summary chart representing types of non-pharmacological and pharmacological treatment methods for PD-associated sleep disorders. (Figure created using BioRender.com; accessed on 2 August 2023).

Although certain drugs and treatments are suggested for sleep disorders, a complete treatment for RLS associated with PD still needs to be achieved. Most commonly, dopamine agonists, calcium-channel alpha-2-delta ligands, clonazepam, and opioids treat RLS [228,229,230]. Depending upon the time of appearance of RLS symptoms, levodopa can be used to treat motor symptoms in PD patients. Also, iron supplementation is considered if ferritin levels are low in the RLS condition [231]. Ropinirole is a nonergoline dopamine agonist used to treat PD and is also used to treat RLS symptoms; it was found to improve overall sleep quality with no adverse drug effects [232]. Some clinical trial studies demonstrated the benefit of treating RLS with levodopa, pergolide, and pramipexole. Low doses of levodopa are enough to treat dopaminergic abnormalities in the case of RLS [233]. A treatment chart representing types of non-pharmacological and pharmacological interventions is given in Figure 5.

## 8. Conclusions

A wide range of sleep disorders may occur as comorbidities with PD. Sleep disorders influence individuals’ physical and mental health and may lead to other psychiatric illnesses. PD-related sleep disorders present as sleep disturbances and are part of underpinning neuropsychological changes, which must be evaluated with great concern. Complex neurochemical networks are the causal factors of sleep disorders and other non-motor complications in PD. Neuronal modeling approaches using experimental animal and computation models of sleep disorders could develop therapeutic stratagems for PD-associated sleep disorders. The scientific evidence demonstrates that circadian disruption accelerates PD-specific degeneration and sleep dysfunction. Deeper knowledge of the link between the circadian rhythm and neurodegeneration is important for the early identification and management of PD-specific neurodegeneration. Studies on the mechanisms of the circadian rhythm and neural circuitries of sleep concerning aging and neurodegeneration are necessary. On the different kinds of therapies for managing circadian dysfunction and regulating the sleep cycle in PD patients, the combination of two or more therapies could be even more promising in attaining positive results and could increase success rates. 

The application of molecular studies at the neuronal level might boost new clinical diagnostic markers specific to PD-associated sleep disorders and circadian-dysfunction-related sleep disorders. The bidirectional link between sleep and the circadian clock must be carefully examined to develop diagnoses and treatments for PD-associated sleep disorders. Depending on the clinical manifestations and underlying pathologies of different sleep disorders in PD, patients could be treated individually according to determined individual variations. Predominantly, symptoms and severity vary per individual. So far, the sparsity of clinical trials in sleep disorders is the major problem in drawing conclusions on the drugs and dosages for sleep disorders in PD.

## Figures and Tables

**Table 1:** Representative studies on sleep disorders associated with Parkinson’s disease.

No.	No. of Subjects	Age	Study Conditions	Study Findings	Ref.
Rapid-eye-movement sleep behavior disorder (RBD)					
1	PD-RBD (*n* = 20); PD without RBD (*n* = 20)	Age- and gender-matched with controls	PD patients with and without RBD were evaluated for neurophysiological abnormalities with single- and paired-pulse TMS, and RMT, CMCT, SP, SICI, and ICF were recorded.	PD-RBD patients showed reduced intracortical facilitation, reduced glutaminergic transmission, and enhanced GABAergic transmission.	[57]
2	PD subjects (*n* = 360); prodromal PD subjects ((*n* = 46); subjects displaying RBD behaviors); controls (*n* = 169)	Mean age: 61.24 years for controls, 61.31 years for PD patients, 68.20 years for prodromal PD subjects	The association of RBD and the level of CSF alpha-synuclein was evaluated.	PD individuals with probable RBD had significantly lower alpha-synuclein levels in CSF. No significant association between daytime sleepiness and CSF alpha-synuclein levels was found.	[61]
3	PD_GBA_ (*n* = 80); PD_GBA-wildtype_ (*n* = 80); controls (*n* = 39)	59 ± 12 years for controls, 64 ± 10 years for PD-GBA patients, 66 ± 10 years for PD-GBA risk-variant patients	PD patients with and without *GBA1* mutation were screened for total CSF alpha-synuclein.	PD_GBA_ patients showed early-onset cognitive decline, high chance of RBD development, and reduced total CSF alpha-synuclein.	[62]
4	Idiopathic RBD patients (*n* = 1061); controls (*n* = 3086)	-	The role of *GBA* variants in the risk of developing idiopathic RBD and development of neurodegeneration was studied.	Individuals with *GBA* variants had increased risk of idiopathic RBD, and the rate of neurodegeneration also increased in *GBA*-variant individuals.	[93]
5	RBD patients (*n* = 261); controls (*n* = 379)	67.2 ± 9.2 years for RBD patients, 58.9 ± 12.3 years for controls	RBD patients and controls were screened for PD-associated SNPs and their effects on RBD and progression of synucleinopathies.	Data from 56 RBD patients showed that 19 developed neurodegeneration during the follow-up period, 9 were diagnosed with PD, and 10 had DLB. The *SCARB2* rs6812193 SNP and the *MAPT* rs12185268 SNP were associated with RBD, and the carriers of these SNPs progressed to synucleinopathies. A few patients with the *USP25* rs2823357 SNP demonstrated faster progression to synucleinopathy from RBD.	[94]
Excessive daytime sleepiness (EDS)					
6	PD patients (*n* = 400)	-	Five-year hospital-based cohort study to analyze the risk factors of EDS in PD using SCOPA-SLEEP-DS scores.	The proportion of EDS in PD increased with longer follow-up. In total, 43% of PD patients had EDS at baseline. A total of 46% of patients without EDS at baseline developed EDS during follow-up.	[65]
7	Unmedicated PD patients (*n* = 423); Controls (*n* = 196)	-	EDS was assessed using the ESS. Clinical, biological, and imaging variables were assessed.	EDS was developed during the follow-up. EDS in PD was associated with autonomic dysfunction, depression, and anxiety. EDS was also associated with presynaptic dopaminergic dysfunction.	[70]
8	Idiopathic PD patients (*n* = 101); unmedicated (*n* = 12); Patients with levodopa monotherapy (*n* = 29); Patients with dopamine agonist monotherapy (*n* = 5); Patients with levodopa plus adjuvant agent therapy (*n* = 55); Patients, who taking anti-depressants (*n* = 26), Patients, who taking benzodiazepines (*n* = 15)	67.3 ± 8.0 years for controls, 65.9 ± 9.5 years for all PD patients, 67.9 ± 9.0 years for PD-RBD patients, and 62.8 ± 9.6 years for PD-non-RBD patients	All patients’ neuropsychological functioning was assessed with standard tests using Wechsler Adult Intelligent Scale-III, Cambridge Neuropsychological Test Automated Battery, and Wechsler Memory Scale-III; daytime sleepiness was assessed with the SCOPA-day, and EDS, with the ESS.	Patients with greater levodopa dose equivalents showed greater nocturnal disturbances and daytime sleepiness but not RBD symptoms. EDS was a significant predictor of slow processing speed, working memory, and verbal frequency performance.	[71]
9	Patients with EDS receiving stable dopaminergic therapy without cognitive impairment or primary sleep disorder (*n* = 31)	-	Safety and efficacy of light therapy on EDS were evaluated. Participants were randomly assigned in a 1:1 ratio to receive bright light and dim light (as control) twice daily in 1-hour intervals for 14 days.	Bright-light therapy significantly improved EDS scores. Bright and dim light improved sleep quality based on the Pittsburg Sleep Quality Index. Bright-light therapy improved mean sleep metrics and sleep quality.	[95]
Insomnia					
10	Drug naïve PD patients (*n* = 182); Controls (*n* = 202).	67.5 ± 9.2 years for patients and 66.2± 9.6 for controls	Participants were assessed for insomnia with the Stavanger Sleepiness Questionnaire and Parkinson’s Disease Sleep Scale before treatment initiation and after 1, 3, and 5 years.	Insomnia prevalence was not higher in PD patients at the 5-year follow-up. Sleep-maintenance problems increased, and solitary-sleep-initiation problems decreased after 5 years.	[75]
11	PD Patients with insomnia randomized for three-arm six-week randomized pilot study (*n* = 18); Placebo (*n* = 6); CBT with BLT (*n* = 6); Doxepin (10 mg/daily) (*n* = 6).	-	This three-arm, six-week randomized pilot study assessed non-pharmacological and pharmacological treatment outputs in PD patients with insomnia. Sleep outcomes were measured using insomnia scales, sleep diaries, actigraphy, and clinical global impression.	Doxepin improved the scores in Insomnia Severity Index, SCOPA-night, and Pittsburgh Sleep Quality Index-Sleep Disturbances Subscale. Doxepin reduced the score on the Fatigue Severity Scale and improved the scores on the Montreal Cognitive Assessment. Non-pharmacological treatment reduced the Insomnia Severity Index.	[96]
12	Patients under 65 received 3 mg eszopiclone or matching placebo at night. Patients 65 or older received 2 mg of eszopiclone or placebo at night (*n* = 30).	35 to 85 years	Patients were equally randomized to eszopiclone and placebo for 6 weeks. Patients with other primary sleep disorders were excluded. Total sleep time, wake after sleep onset, and number of awakenings were measured.	Significant differences were found in the number of awakenings, sleep quality, and wake after sleep onset, favoring eszopiclone. Eszopiclone did not increase the total sleep time but improved the sleep quality compared with the placebo group.	[97]
Obstructive sleep apnea (OSA)					
13	PD patients with OSA (*n* = 67).	64.7 years	Patients were treated with CPAP, and motor symptoms were assessed using the MDS-UPDRS and TUG with a follow-up time of 3, 6, and 12 months.	CPAP treatment stabilized the motor function over 12 months of follow-up treatment.	[84]
14	PD patients (*n* = 239); PD (*n* = 66) with OSA including mild (*n* = 34), moderate (*n* = 16), severe sleep apnea (*n* = 16); PD without OSA (*n* = 173).	*n* = 66 PD patients with OSA had a mean age of 45 years; *n* = 173 PD patients without OSA had a mean age of 81 years	Participants underwent assessments to examine disease severity, polysomnography characteristics, and non-motor symptoms.	Binary logistic regression analysis showed that age and male gender were risk factors for OSA. RBD and higher levodopa equivalent dose were protective factors against OSA. Thus, OSA in PD was lower in PD patients with RBD. And OSA could increase excessive day sleeping in PD patients.	[92]
15	Subjects were divided into OSA and non-OSA groups (*n* = 95).	69.1 ± 3.4 years	Subjects were evaluated with protocols that included polysomnography, BMR, and body composition. BMR was evaluated in the morning after polysomnography.	Patients with OSA had higher values in weight, fat mass, arousal, and AHI. The OSA group had lower REM sleep.	[98]
16	Idiopathic PD patients (*n* = 67)	Mean age of 64.4 years	Idiopathic PD patients were recruited from a movement-disorder clinic. OSA was defined using the AHI. The H&Y Scale and MDS-UPDRS were used to assess PD severity. And NMSs were assessed with the MoCA, ESS, Fatigue Severity Scale, Apathy Scale, BDI, HDAS, and PDSS.	OSA in PD was associated with sleepiness and cognitive dysfunction. Treatment for OSA could improve excessive sleepiness and cognitive dysfunction in PD.	[99]
Restless legs syndrome (RLS)					
17	PD patients with *parkin* mutations (*n* = 11); Sex matched IPD patients (*n* = 11)	PD patients with *parkin* mutations were aged 35–60 years and were from seven families; IPD patients were aged 51–65 years.	Patients with *parkin* mutations and IPD patients were compared to evaluate the sleep–wake phenotype using the UPDRS, ESS, MMSE, and RLS Study Group Rating Scale; a sleep specialist interview; and video-polysomnography.	*Parkin* patients showed sleep phenotypes like insomnia and RLS, and neuronal loss. Parkin-mutation patients had all polygraphical abnormalities reported in IPD. Two *Parkin* siblings had central hypersomnia and normal night-time sleep.	[100]
18	PD patients (*n* = 74); Drug-I patients (*n* = 16); Patients treated with levodopa/aromatic L-amino acid decarboxylase inhibitor, monoamine oxidase B inhibitor and amantadine (*n* = 58)	65.5 ± 9.1 years	Patients were assessed for RLS based on the diagnostic criteria of the International RLS Study Group revised in 2003.	The frequency of RLS in PD patients was higher than the general RLS population. PD patients with RLS had worse sleep quality, anxiety, depression, and autonomic disturbances.	[101]
19	Idiopathic PD patients (*n* = 108); Matched controls (*n* = 424)	≥35 years	This comparative study analyzed the prevalence of RLS in PD patients and investigated the quality of life, nutritional status, and clinical characteristics using IRLSSG, PD severity scales, psychiatric features, nutritional status, and quality of life.	RLS was significantly more common in IPD patients than controls. PD patients with RLS suffered from more anxiety, and worse nutritional status and quality of life. RLS was found to be correlated with psychiatric problems and cognitive impairment.	[102]
20	PD patients (*n* = 225)	-	RLS was diagnosed using IRLSSG criteria. Orthostatic vital signs and blood pressure were monitored.	PD patients with RLS showed nocturnal/supine hypertension and fluctuations in blood pressure and some sleep dysfunctions. RLS could be a determinant for neurocirculatory abnormalities.	[103]
21	Drug naïve early, unmedicated PD patients (*n* = 200); Controls (*n* = 173)	Age- and gender-matched controls	Subjects were assessed for RLS with structured interviews, clinical examinations, and blood samples. RLS was diagnosed using IRLSSG criteria.	PD patients reported leg restlessness, which was 3-fold greater in patients than in controls, which could indicate a relative risk for RLS.	[104]

PD: Parkinson’s disease; RBD: rapid-eye-movement sleep behavior disorder; RMT: resting motor threshold; CMCT: central motor conduction time; SP: silent period; SICI: short-interval intracortical inhibition; ICF: intracortical facilitation; PD_GBA_: PD patients with mutation in the glucocerebrosidase (*GBA1*) gene; PD_GBA-wildtype_: PD patients without *GBA1* mutation; CSF: cerebrospinal fluid; DLB: dementia with Lewy bodies; EDS: excessive daytime sleepiness; SCOPA-SLEEP-DS: Scales for Outcomes in PD-Sleep Scale-Daytime Sleepiness; SPECT: single-photon-emission computed tomography; ESS: Epworth Sleepiness Scale; SCOPA-day: Scales for Outcomes in PD (sleep scale to measure general daytime sleepiness); CPAP: continuous positive airway pressure; AHI: Apnea–Hypopnea Index; MDS-UPDRS: Movement Disorder Society-Sponsored Unified Parkinson’s Disease Rating Scale; TUG: Timed Up and Go; H&Y Scale: Hoehn–Yahr Scale; MoCA: Montreal Cognitive Assessment; ESS: Epworth Sleepiness Scale; BDI: Beck Depression Inventory; HDAS: Hospital Depression and Anxiety Scale; PDSS: Parkinson’s Disease Sleep Scale; RLS: restless legs syndrome; MMSE: Mini-Mental State Examination; IRLSSG: International Restless Legs Syndrome Study Group.

**Table 2:** Highlights of the genetic heterogeneity of sleep disorders in patients with PD.

S. No.	No. of Subjects	Age, Gender, and Other Details of the Subjects	Study Conditions	Study Findings	Ref.
1	Patients with early-onset PD (*n* = 124); Patients completed the assessments (*n* = 84).	Age at onset of 34.1 ± 5.7 years. Male-to-female ratio was 66:58.	Native Korean patients with early-onset PD clinically examined for PD according to UKPDSBB criteria.	Among 84 patients, 23 carried *Parkin* mutations. Further, 1 patient was homozygote; 13 patients were heterozygotes; and 6 patients were single heterozygotes. Among 13 heterozygotes, 11 had exon rearrangements, 2 carried point mutations (p.Gly284Arg with exon 2-3-4 del, p.Leu272Ile, and p.Ala398Thr), and 1 had a frameshift mutation (p.His200ThrfsX6 with exon 4 del).	[167]
2	Patients with familial parkinsonism (*n* = 106)	The two groups (G2019S-mutation carriers and non-carriers) of patients had similar age at onset and age at examination.	Patients were clinically and genetically evaluated for *LRRK2* G2019S mutation and underwent cognitive and neuropsychiatric testing.	G2019S mutation was identified in 34 out of 106 patients. A total of 71 patients gave consent for cognitive and neuropsychiatric testing. Among 71 patients (45 men, 26 women), 23 (11 men, 12 women) were G2019S-mutation carriers, and 48 (34 men, 14 women) were non-carriers. Cognitive functions were similarly affected in both carriers and non-carriers. Behavioral abnormalities, depression, and hallucinations were frequent in *LRRK2* G2019S carriers.	[168]
3	Idiopathic RBD patients of European ancestry (*n* = 265); Controls of European origin, including 189 controls who did not have PD at recruitment (*n* = 2240); 120 subjects formed an independent PD cohort, including 120 Ashkenazi-Jewish patients from Tel-Aviv, Israel.	The cohort had 79.6% men, age at enrollment of 67.2 ± 9.8 years.	Patients were diagnosed according to the International Classification of Sleep Disorders (ICSD-2) criteria. The independent PD cohort was analyzed for founder *GBA* mutations, and 5 were screened for RBD using the RBD Screening Questionnaire (RBDSQ).	*GBA* mutations were significantly more frequent among RBD patients. In the cohort of 120 patients, 19 were *GBA* mutation carriers. Of these 19, 9 patients had RBD. The results demonstrate that rapid-eye-movement sleep behavior disorder is associated with *GBA* mutations.	[169]
4	RBD patients (*n* = 261); Controls (*n* = 379)	Of the RBD patients, 80% were men, aged 67.2 ± 9.2 years; data on gender and age were available for 250 and 142 individuals, respectively. Of the controls, 50% were men, aged 58.9 ± 12.3 years, and data on gender and age available were for 369 and 183 individuals, respectively.	All the RBD patients were recruited through the International RBD Study Group and were diagnosed with RBD according to the International Classification of Sleep Disorders (ICSD-2) criteria.	Before adjusting for sex and age variables, the *SCARB2* rs6812193 SNP was associated with RBD with odds ratio of 0.67 and 95% confidence interval. After adjusting for sex and age variables, the *SCARB2* rs6812193 SNP and the *MAPT* rs12185268 SNP were associated with RBD with odds ratio of 0.23 and 95% confidence interval. Data for progression from RBD to synucleinopathies (*n* = 56) showed that 7 carriers of the *MAPT* rs12185268 SNP progressed to synucleinopathy, 11 carriers of the *SCARB2* rs6812193 SNP progressed to synucleinopathy, a few patients with the *USP25* rs2823357 SNP in the recessive model demonstrated faster progression from RBD to any synucleinopathy.	[152]
5	Individuals with early-onset PD. *GBA* carrier (*n* = 33); Non-carriers (*n* = 114).	Age at onset of PD < 51 years	Participants were screened for mutations in *SNCA*, *PARKIN*, *PINK-1*, *DJ-1*, *LRRK2*, and *GBA*. Given the higher frequency of *GBA* mutations among Ashkenazi Jews, participants who self-reported Ashkenazi-Jewish ancestry were further screened for an additional 6 common *GBA* mutations (V394L, D409G, A456P, R496H, 84GG, and exon 2 IVS2 + 1) with direct sequencing.	Among 147 participants, 33 were *GBA* carriers, while 60 did not have any mutations in the other genes tested. Among 33 *GBA* mutation carriers, 7 were heterozygous L444P carriers; a total of 16 heterozygous were N370S carriers; only 1 was N370S homozygote; in total, 2 were 84GG carriers; and 1 was an R496H carrier. And 3 had both *GBA* and *PARKIN* mutations, while 3 had both *GBA* and *LRRK2* G2019S mutations.	[170]
6	PD patients (*n* = 1893)	A total of 142 PD cases had a variant detected in the *GBA* gene, and their mean age was 65.6 years. Cases carrying GD-causing variants in the *GBA* gene were younger (mean age of 62.9 years and non-carriers mean age of 67.6 years.	The *GBA* gene was fully sequenced, and cognitive and motor features were assessed using the MoCA and MDS-UPDRS part 3.	In total, 48 were heterozygous carriers for Gaucher’s disease, while 117 had non-synonymous variants, previously associated with PD, and patients carried variants of the *GBA* gene of unknown significance. *L444P* was the most common pathogenic *GBA* mutation. Patients with *GBA* mutations were more likely to present with PIGD and showed advanced scores on the H&Y Scale. In the early disease stage, there were no differences in cognitive function between carriers and non-carriers of *GBA* mutation.	[148]
7	Patients with iRBD (*n* = 1061); Controls (*n* = 3086)	Controls had a mean age of 46.5 ± 15.0 years, and 46.6% were men; patients had a mean age of 60.5 ± 9.9 years, and 81% were men.	*GBA* was fully sequenced using molecular inversion probes and Sanger sequencing.	In total, 9.5% of iRBD patients and 4.1% of controls had *GBA* variants. The mild p.N370S variant of *GBA* was found in 1.9% of iRBD patients and 0.5% of controls. Severe variants, like p.L444P, p.D409H, p.W291X, p.H255Q, and p.R131L, were found in 0.6% of iRBD patients and in 0.03% of controls. The p.E326K variant was associated with iRBD (4.4%) and controls (1.5%). The carrier frequency of variant p.T369M was only slightly elevated in iRBD patients (1.9%) compared with controls (1.7%) without any statistical significance.	[149]
8	*n* = 158 LRRK2 patients, *n* = 80 GBA and *n* = 361 sporadic-PD participants	Mean age: LRRK2 PD participants, 63.8 ± 9.2; GBA PD, 62.7 ± 9.9; sPD, 63.8 ± 9.7.	Participants were evaluated annually with a battery of motor and non-motor scales and 123-I Ioflupane DAT imaging. Genetic testing for *LRRK2* (for G2019S, R1441G/C, and N1437H mutations) and *GBA* (N370S, L483P, L444P, IVS2 + 1, and 84GG mutations) were performed.	Compared with sPD patients, GBA PD patients were likely women. In total, 89% of GBA patients carried the N370S mutation. Among LRRK2 patients, 89% carried the G2019S mutation. DAT imaging results showed higher specific binding ratios in contralateral putamen and putamen in both LRRK2 and GBA PD patients compared with sPD patients. LRRK2 had higher hyposmic scores. GBA PD patients had higher RBDSQ scores. MoCA scores or neurocognitive battery scores showed no difference among the groups.	[153]
9	PD patients categorized into four groups: LRRK2 PD (*n* = 80); GBA PD (*n* = 78); GBA-LRRK2 PD (*n* =12); and mutation-negative PD (*n* = 80).	-	Odds ratios were estimated using published data on frequencies of GBA and LRRK2 mutations. Clinical data were collected from medical records.	Probable RBD was significantly more common in GBA-PD than in LRRK2-PD. None of the GBA-LRRK2-PD patients reported RBD. Compared with LRRK2 and MNPD participants, GBA PD patients showed significantly high dementia. And psychosis was the most common in GBA-PD and the least common in LRRK2-GBA-PD.	[154]
10	2764 unrelated consecutive PD patients, of which 123 were *GBA* carriers (67 mild-p.N370S and 56 severe p.L444P) and 2641 were non-carriers.	-	Brain perfusion and DAT imaging analysis were performed, including dementia and DLB.	GBA carriers were at greater risk of dementia and death than PD non carriers. GBA carriers had worse motor symptoms, and reduced posterior parietal and occipital cortical synaptic activity, and nigrostriatal function than PD non-carriers.	[14]

*GBA*: glucocerebrosidase; *LRRK 2*: leucine-rich repeat kinase 2; ICSD: International Classification of Sleep Disorders; UKPDSBB: United Kingdom Parkinson’s Disease Society Brain Bank; PIGD: postural instability and gait difficulty; H&Y Scale: Hoehn–Yahr scale; MoCA: Montreal Cognitive Assessment; MDS-UPDRS: Movement Disorder Society-Sponsored Unified Parkinson’s Disease Rating Scale; sPD: sporadic Parkinson’s disease; DAT imaging: dopamine (DA) transporter imaging; RBDSQ: rapid-eye-movement (REM) sleep behavior disorder (RBD) Screening Questionnaire; SNP: single nucleotide polymorphism; MNPD: mutation-negative Parkinson’s disease; DLB: dementia with Lewy bodies.

## Data Availability

Not applicable.

## Abbreviations

PD	Parkinson’s disease
iPD	idiopathic Parkinson’s disease
AD	Alzheimer’s disease
CNS	Central nervous system
REM	Rapid eye movement
NREM	Non-rapid eye movement
RBD	Rapid eye movement sleep behavior disorder
iRBD	idiopathic Rapid eye movement sleep behavior disorder
EDS	Excessive daytime sleeping
RLS	Restless leg syndrome
OSA	Obstructive sleep apnea
SRBD	Sleep-related breathing disorder
CRSWD	Circadian rhythm sleep–wake disorder
DSWPD	Delayed sleep–wake phase disorder
α-SYN	Alpha-synuclein
SCNA	Alpha-synuclein
PARK2	Parkin 2
LRRK2	Leucine-rich repeat kinase 2
PINK	PTEN-induced putative kinase
PRKN	Parkin RBR E3 ubiquitin-protein ligase
GBA	Glucocerebrosidase genes
ATP13A2	ATPase type 13A2
ICSD	International Classification of sleep disorders
CSF	Cerebrospinal fluid
LBD	Lewy body dementia
SNPc	Substantia nigra pars compacta
SN	Substantia nigra
VTA	Ventral tegmental area
PVN	Paraventricular nucleus
MPN	Medial preoptic nucleus
MPTP	1-methyl-4-phenyl-1,2,3,6-tetrahydropyridine
DA	Dopamine
GABA	Gamma-aminobutyric acid
IL-1β	Interleukin-1β
TNF-α	Tumour necrosis factor- α
ESS	Epworth sleepiness scale
DaTSCAN	Dopamine transporter scan
CLOCK	Circadian locomotor output cycles kaput
HCRT2-SAP	Hypocretin-2-sap
CPAP	Continuous positive airway pressure
EMG	Electromyographic
MoCA	Montreal cognitive assessments
MMSE	Mini-mental State Examination
D2R	Dopamine 2 receptor
PLMS	Periodic leg movements in sleeping
ALS	Amyotrophic lateral sclerosis
ARAS	Ascending reticular activating system
SCN	Suprachiasmatic nucleus
PER3	Period circadian regulator 3
CRY1	Cryptochrome
Nr1d1	Nuclear receptor subfamily 1 group D member 1
Nr1d2	Nuclear receptor subfamily 1 group D member 2
PDSS	Parkinson’s disease sleep scale
SCOPA	Scales for outcomes in PD sleep
PSQI	Pittsburgh sleep quality index
RBDSQ	RBD screening questionnaire
RBD-I	Innsbruck RBD inventory
RBDQ-HK	Hong Kong RBD questionnaire
RBD-IQ	RBD-single question screen
MSGBs	Minor salivary gland biopsies
CSHQ	Cleveland sleep habits questionnaire
MRPI	Magnetic resonance parkinsonism index
MRI	Magnetic resonance imaging
DTI	Diffusion tensor imaging
fMRI	Functional magnetic resonance imaging
FA	Fractional anisotropy
TMS	Transcranial magnetic stimulation
DAT-SPECT	Dopamine transporter single-photon emission computed tomography
BLT	Bright light therapy
CBT	Cognitive behavioral therapy
PBM	Photobiomodulation
tDCS	Transcranial direct current stimulation
LCIG	Levodopa/carbidopa intestinal gel
LCE	Levodopa/carbidopa/entacapone
